# Light‐dependent N‐terminal phosphorylation of LHCSR3 and LHCB4 are interlinked in *Chlamydomonas reinhardtii*

**DOI:** 10.1111/tpj.14368

**Published:** 2019-05-30

**Authors:** Martin Scholz, Philipp Gäbelein, Huidan Xue, Laura Mosebach, Sonja Verena Bergner, Michael Hippler

**Affiliations:** ^1^ Institute of Plant Biology and Biotechnology University of Münster Schlossplatz 8 Münster 48143 Germany; ^2^ Present address: Max Planck Institute of Molecular Plant Physiology Am Mühlenberg 1 Potsdam‐Golm 14476 Germany

**Keywords:** photosynthesis, light harvesting, high light stress, protein phosphorylation

## Abstract

Phosphorylation dynamics of LHCSR3 were investigated in *Chlamydomonas reinhardtii* by quantitative proteomics and genetic engineering. LHCSR3 protein expression and phosphorylation were induced in high light. Our data revealed synergistic and dynamic N‐terminal LHCSR3 phosphorylation. Phosphorylated and nonphosphorylated LHCSR3 associated with PSII‐LHCII supercomplexes. The phosphorylation status of LHCB4 was closely linked to the phosphorylation of multiple sites at the N‐terminus of LHCSR3, indicating that LHCSR3 phosphorylation may operate as a molecular switch modulating LHCB4 phosphorylation, which in turn is important for PSII‐LHCII disassembly. Notably, LHCSR3 phosphorylation diminished under prolonged high light, which coincided with onset of CEF. Hierarchical clustering of significantly altered proteins revealed similar expression profiles of LHCSR3, CRX, and FNR. This finding indicated the existence of a functional link between LHCSR3 protein abundance and phosphorylation, photosynthetic electron flow, and the oxidative stress response.

## Significance Statement

We analyzed light‐dependent phosphorylation of LHCSR3 and LHCB4 by quantitative proteomics, genetic engineering, and functional measurements. N‐terminal LHCSR3 phosphorylation is light dependent and modulates phosphorylation of LHCB4 and the association of LHCSR3 with photosynthetic multi‐protein complexes. Our data revealed the functional importance of LHCSR3 phosphorylation, pointing to unforeseen functions in algal photosynthesis.

## Introduction

Oxygenic photosynthesis is driving the conversion of solar energy into chemical energy. This energy is used for carbon dioxide assimilation, leading to the formation of complex organic material. The light to energy conversion is catalyzed by two photosystems (PSI and PSII), which are embedded in the thylakoid membrane. The cytochrome *b*_*6*_ */f* complex (cyt *b*_*6*_ */f* complex) interconnects photosynthetic electron transfer reactions between the two photosystems and participates in the translocation of protons into the thylakoid lumen. The photosynthetic electron transfer leads to light‐dependent water oxidation, NADP^+^ reduction, and ATP formation (Whatley *et al*., 1963). The formation of ATP is catalyzed by the fourth, large multi‐protein complex in the thylakoid membrane, ATP synthase, that uses the proton‐motive force that is generated by the light reactions (Mitchell, 1961). The Calvin−Benson−Bassham cycle (Bassham *et al*., 1950) utilizes ATP and NADPH, produced via linear electron flow (LEF), to fix CO_2_. Alternatively, ATP synthesis can be driven by cyclic electron flow (CEF) operating between PSI and the cyt *b*_*6*_ */f* complex (Arnon, 1959). This is important as CEF provides additionally required ATP for CO_2_ fixation (Lucker and Kramer, 2013). The onset of CEF is likely to be linked to the formation of a PSI‐cyt *b*_*6*_ *f* complex (Iwai *et al*., 2010; Steinbeck *et al*., 2018).

Lumen acidification via CEF is also required for acclimation to excess light (Munekage *et al*., 2004; Dang *et al*., 2014; Johnson *et al*., 2014; Kukuczka *et al*., 2014) by promoting the energy‐dependent component (qE) of nonphotochemical quenching (NPQ). qE, the fastest component of NPQ, drives the thermal dissipation of excess absorbed light energy and therefore provides effective photo‐protection. PSBS, a PSII subunit, is mechanistically required for qE in vascular plants (Li *et al*., 2000). In contrast, qE is mainly facilitated by LHCSR3 in the green alga *Chlamydomonas reinhardtii* (Peers *et al*., 2009). In addition LHCSR3, LHCSR1 is another member of an ancient light‐harvesting protein family that is absent in vascular plants and active in qE in the alga (Peers *et al*., 2009). LHCSR3 is rapidly induced in expression after the onset of light (Naumann *et al*., 2007; Peers *et al*., 2009). LHCSR3 is pH‐responsive and converts into a dissipative state under low pH conditions as shown by *in vitro* and *in vivo* experiments (Bonente *et al*., 2011; Liguori *et al*., 2013; Ballottari *et al*., 2016). Recently, PSBS function has also been analyzed in *C. reinhardtii*, in which it transiently accumulates in thylakoid membranes after the onset of light (Correa‐Galvis *et al*., 2016; Tibiletti *et al*., 2016). However, its exact role in the qE mechanism of the alga remains elusive. Interestingly, expression of PSBS and LHCSR1 is controlled through UV‐B perception via UVR8 (Allorent *et al*., 2016; Tilbrook *et al*., 2016). LHCSR3 expression, conversely, is controlled through blue‐light perception via phototropin (Petroutsos *et al*., 2016), calcium, the chloroplast CAS protein and photosynthetic electron transfer (Petroutsos *et al*., 2011; Maruyama *et al*., 2014). LHCSR3 associates with PSII–LHCII supercomplexes in which three LHCII trimers are attached to each side of the core (C_2_S_2_M_2_L_2_) (Tokutsu and Minagawa, 2013). The PSBR subunit of PSII is required for efficient binding of LHCSR3 to the PSII–LHCII supercomplexes (Xue *et al*., 2015), suggesting that LHCSR3 is in close contact with the minor antenna proteins LHCB4 (CP29) and LHCB5 (CP26) (Semchonok *et al*., 2017). The two monomeric LHCII proteins CP26 and CP29 and the major LHCII protein LHCBM5 (Takahashi *et al*., 2006), as well as LHCSR3 (Allorent *et al*., 2013), have been suggested to migrate during state transitions. State transitions also contribute to NPQ (qT) and are involved in balancing excitation energy between PSI and PSII (Eberhard *et al*., 2008). The initiation of state transitions requires phosphorylation of LHC proteins. Phosphorylated LHCII proteins disconnect from PSII and move towards PSI‐LHCI (state 2). The amount of mobile LHCII antenna is about 80% in *C. reinhardtii* (Delosme *et al*., 1996). However, the extent of PSI antenna size increase is currently under debate as well as whether light energy is mainly dissipated or redistributed between the two photosystems (Iwai *et al*., 2010; Nagy *et al*., 2014; Unlu *et al*., 2014; Nawrocki *et al*., 2016). The process of state transitions can be reversed when LHCII proteins are de‐phosphorylated and re‐associate with PSII (state 1). The STT7 kinase in *C. reinhardtii* (or its ortholog STN7 in *Arabidopsis thaliana*) catalyzes the phosphorylation of the LHCII proteins. Deletion of the kinase prevents initiation of state transitions (Depege *et al*., 2003; Bellafiore *et al*., 2005). Under physiological conditions, the redox poise of the plastoquinone pool modulates the activity of the STT7 kinase (Vener *et al*., 1997; Zito *et al*., 1999). At the same time, dephosphorylation of the LHCII proteins may occur via a PP2C‐type phosphatase (Pribil *et al*., 2010; Shapiguzov *et al*., 2010). In addition the phosphorylation of LHCII proteins, the STT7 kinase also phosphorylates LHCSR3 (Bergner *et al*., 2015). Notably, phosphopeptides stemming from the N‐terminal and C‐terminal regions of LHCSR3 were only detected when cells were cultivated in high light (Bergner *et al*., 2015) as LHCSR3 expression is strongly induced in *C. reinhardtii* under such stress conditions (Peers *et al*., 2009). In the absence of STT7, no N‐terminal phosphorylation of LHCSR3 was detectable (Bergner *et al*., 2015). In contrast, the C‐terminal phosphorylation of LHCSR3 was not affected by deletion of STT7 (Bergner *et al*., 2015).

Association of LHCSR3 with the putative CEF supercomplex, comprised of PSI‐LHCI, LHCII, the cyt *b*_*6*_ */f* complex and ferredoxin (Fd)‐NADP^+^ oxidoreductase (FNR) (Iwai *et al*., 2010), was increased in the absence of STT7 (Bergner *et al*., 2015). Besides the association of LHCSR3 with PSII–LHCII complexes, there is also increasing evidence that LHCSR3 associates with PSI−LHCI complexes (Allorent *et al*., 2013; Tokutsu and Minagawa, 2013; Bergner *et al*., 2015; Pinnola *et al*., 2015; Xue *et al*., 2015). However, it is currently unknown how the binding of LHCSR3 to the photosynthetic multi‐protein complexes is regulated.

Here, we quantified abundances of LHCSR3 phosphorylation and investigated its functional role(s). We used site‐directed mutagenesis and nuclear transformation of *C. reinhardtii* to alter the N‐terminal LHCSR3 phosphorylation sites to investigate their impact on overall LHCSR3 phosphorylation, on association of LHCSR3 with the photosynthetic multi‐protein complexes and on phosphorylation of LHCB4.

## Results

The quantification of protein phosphorylation in experimental settings is analytically challenging, but it is an important endeavor to relate phosphorylation quantities to functional consequences. To resolve dynamic protein phosphorylation in response to high light stress, *C. reinhardtii* wild‐type (WT) cells were grown under photoautotrophic conditions at low light and shifted to high light intensities of 200 and 500 μmol m^−2^ sec^−1^. For quantitation of phosphorylation sites, peptide abundances were determined by parallel reaction monitoring (PRM) analyzing samples 0, 1, 4, and 24 h after the high light shift. We focused on phosphorylation dynamics and total protein abundances of LHCSR3 and LHCB4. Additionally, samples derived from cells grown under 500 μmol photons m^−2^ sec^−1^ were analyzed by LC‐MS/MS using a nontargeted approach (data‐dependent acquisition (DDA)) for subsequent label‐free protein quantification in MaxQuant (Cox and Mann, 2008). In order to confirm abundances determined using mass spectrometry, selected proteins were analyzed by immunoblotting.

### N‐terminal phosphorylation of LHCSR3 reveals a dynamic pattern

**Figure 1:**
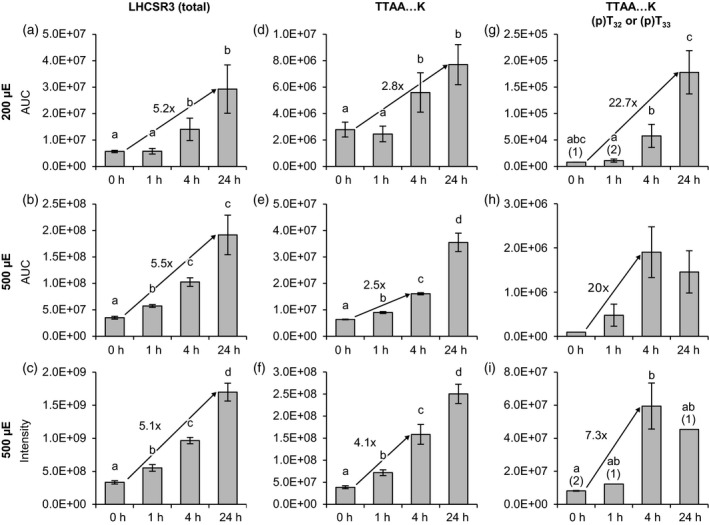
Changes in LHCSR3 abundance and phosphorylation (T32/T33) in response to different light conditions. Algal cultures were exposed to 200 μmol photons m^−2^ sec^−1^ (200 μE; a, d, g) or 500 μmol photons m^−2^ sec^−1^ (500 μE; b, e, h) highlight in HS medium and samples were taken at the indicated time points. Label‐free quantification was performed by parallel reaction monitoring (PRM, top and middle rows). Peptide and protein abundances are represented as ‘areas under curve’ (AUC). The AUCs of two proteotypic peptides were summed to reflect total LHCSR3 abundance. For the 500 μmol photons m^−2^ sec^−1^ experiment supplementary quantitative data was obtained by label‐free quantification using the MaxLFQ algorithm (c, f, i). (a−c) Total LHCSR3. (d−f) PeptideT_32_T_33_AAEPQTAAPVAAEDVFAYT, nonphosphorylated. (g−i) Peptide (p)T_32_(p)T_33_AAEPQTAAPVAAEDVFAYT, singly phosphorylated at either T_32_ or T_33_. Data represent mean ± standard deviation (200 μmol photons m^−2^ sec^−1^: *n* = 4; 500 μmol photons m^−2^ sec^−1^ (PRM): *n* = 3 (0 h: *n* = 2), 500 μmol photons m^−2^ sec^−1^ (MaxLFQ): *n* = 3). Numbers in parentheses indicate the number of data points if low abundances did not permit quantification in all replicates. Welch's *t*‐test (unpaired, two‐tailed) was used to analyze the data: Values labelled with identical letters, or no letters, do not show statistically significant differences (*P* > 0.05) (p), phosphorylated residue. Only kinetics of protein abundance changes, but not absolute protein or peptide levels are comparable between the two high light experiments, since samples were analyzed with different LC‐MS configurations.

The N‐terminus of LHCSR3 is phosphorylated in an STT7‐dependent manner, while the C‐terminal phosphorylation of LHCSR3 is STT7‐independent (Bergner *et al*., 2015). N‐terminal phosphorylation occurs at S26, S28, T32, and T33 of LHCSR3. We quantified the amounts of total LHCSR3 as well as of the nonphosphorylated and the singly phosphorylated isoforms of the peptide T_*32*_T_*33*_AAEPQTAAPVAAEDVFAYTK (Figure 1, Appendices [Link], [Link], [Link]). LHCSR3 expression was induced about fivefold 24 h after the onset of high light treatment with 200 or 500 μmol photons m^−2^ sec^−1^ (Figures 1a−c and S1). Single phosphorylation at T32 or T33 was almost undetectable at 0 h, but increased more than total LHCRS3 protein with maxima after 24 and 4 h corresponding to an about 20‐fold increase when subjected to 200 and 500 μmol photons m^−2^ sec^−1^, respectively (Figure 1g−i). The differences in the fold change of total LHCSR3 and the phosphopeptide indicate that relative phosphorylation levels increased in response to high light. The latter assumption is supported by the fact that the amounts of the nonphosphorylated counterpart of this peptide increased more slowly than total LHCSR3 until phosphorylation reached peak levels after 24 h (200 μmol photons m^−2^ sec^−1^) and 4 h (500 μmol photons m^−2^ sec^−1^) according to PRM measurements (Figure 1d,e). Double phosphorylation of T32 and T33 is likely to occur, yet we were not able to quantify corresponding peptides.

We also attempted to quantify singly, doubly, and triply phosphorylated versions of the LHCSR3 peptide S_26_VS_28_GRRT_32_T_33_AAEPQTAAPVAAEDVFAYTK (Figure S2, Appendices [Link], [Link]) by PRM. It is important to mention that the PRM experiments did not allow for the discrimination of phosphoisoforms. Therefore, amounts given for singly or multiply phosphorylated peptides are likely to represent the totals of several co‐eluting isoforms. Under both light regimes, the corresponding peptides were virtually absent at 0 h and after 1 h high light. While maximal abundances of the peptides were detected after 24 h in samples from cells kept under 200 μmol photons m^−2^ sec^−1^, abundances showed a tendency to reach peak levels already after 4 h and decreased until 24 h under 500 μmol photons m^−2^ sec^−1^ (Figure S2), similar to the phosphorylated peptide T_*32*_T_*33*_AAEPQTAAPVAAEDVFAYTK described above.

### Quantitation of the high light stress responses

**Figure 2:**
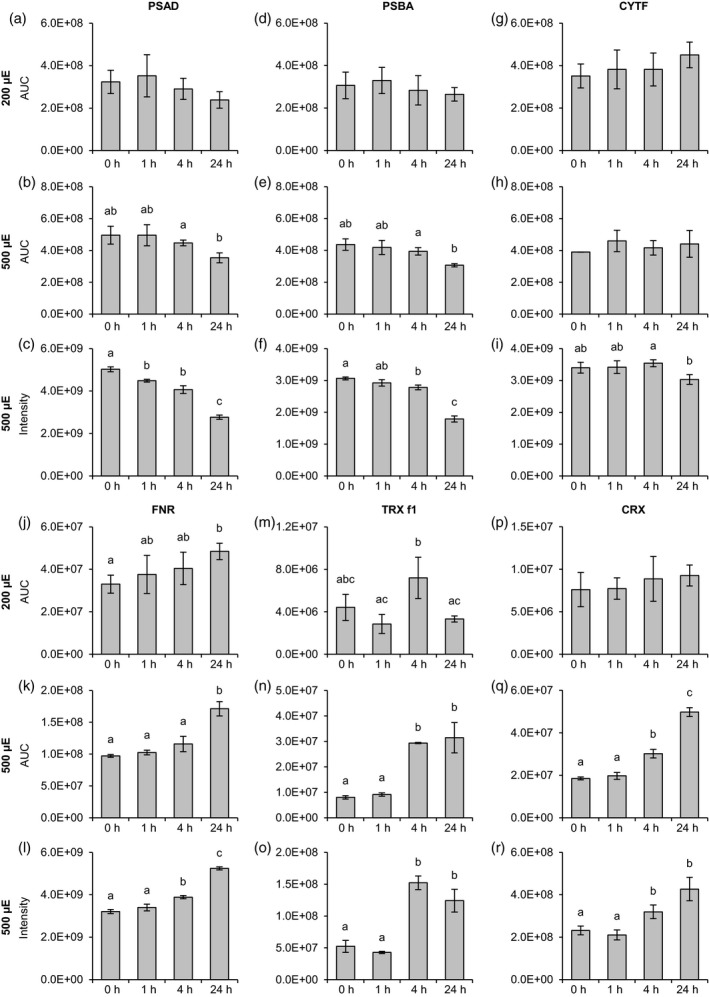
Changes in the abundances of photosynthetic and redox‐related proteins in response to different light conditions. Algal cultures were exposed to 200 μmol photons m^−2^ sec^−1^ (200 μE; a, d, g, j, m, p) or 500 μmol photons m^−2^ sec^−1^ (500 μE; b, e, h, k, n, q) highlight and samples were taken at the indicated time points. Label‐free quantification was performed as described in Figure 1. For the 500 μmol photons m^−2^ sec^−1^ experiment supplementary quantitative data was obtained by label‐free quantification using the MaxLFQ algorithm (c, f, i, l, o, r). (a−c) Photosystem I reaction center subunit II (PSAD). (d−f) Photosystem II protein D1 (PSBA). (g−i) Cytochrome f (CYTF). (j−l) Ferredoxin:NADP+ oxidoreductase (FNR). (m−o) Thioredoxin f1 (TRX f1). (p−r) Calredoxin (CRX). Data represent mean ± standard deviation (200 μmol photons m^−2^ sec^−1^ : *n* = 4; 500 μmol photons m^−2^ sec^−1^ (PRM): *n* = 3 (0 h: *n* = 2), 500 μmol photons m^−2^ sec^−1^ (MaxLFQ): *n* = 3). Welch's *t*‐test (unpaired, two‐tailed) was used to analyze the data: Values labelled with identical letters, or no letters, do not show statistically significant differences (*P* > 0.05). (p), phosphorylated residue. Only kinetics of protein abundance changes, but not absolute protein or peptide levels are comparable between the two high light experiments, since samples were analyzed with different LC‐MS configurations.

Parallel reaction monitoring quantitation (Figure 2 and Appendices S1 and ;S2) revealed no significant change in PSI and PSII quantities after 24 h under 200 but a decrease in the quantities of both photosystems under 500 μmol photons m^−2^ sec^−1^ as measured by the amount of photosystem I reaction center subunit II (PSAD) and photosystem II P680 reaction center D1 (PSBA) peptides (Figure 2a–f). In contrast, the amount of cyt *b*_*6*_ */f* complex was stable under both light conditions as indicated by quantitation of CYTF peptides (Figure 2g–h). Moreover, FNR levels were elevated, particularly after 24 h under 500 μmol photons m^−2^ sec^−1^ (Figures 2j–l and S1). The amounts of TRX*f1* and CRX were significantly increased when comparing the 0 and 24 h timepoints under 500 μmol photons m^−2^ sec^−1^, whereas under 200 μmol photons m^−2^ sec^−1^, no significant differences in abundances were observable (Figure 2m–r; Figure S1). As CRX is an efficient electron donor to PRX1 (Hochmal *et al*., 2016), which is driving detoxification of hydrogen peroxide, enhanced reactive oxygen species (ROS) stress can be anticipated under 500 μmol photons m^−2^ sec^−1^.

**Figure 3:**
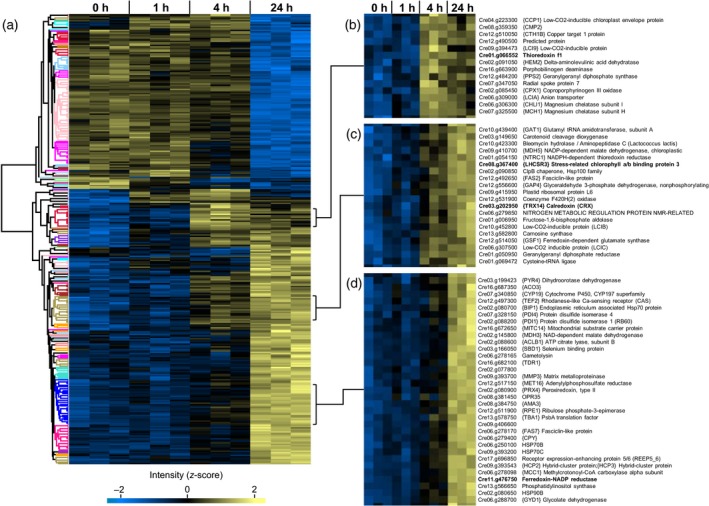
Temporal changes of protein levels in response to high light (500 μmol photons m^−2^ sec^−1^). Quantitative data was obtained from non‐targeted (DDA) LC‐MS/MS experiments. Clustering (average linkage, Euclidean distance) is based on normalized and z‐scored log2 protein intensities. Each lane represents one biological replicate. (a) Hierarchical cluster analysis of 327 proteins with significantly (*P* < 0.01, anova) altered abundances. (b–d) Magnified views of subclusters containing TRXf1 (b), LHCSR3/CRX (c) and FNR(d).

To assess overall sample quality and to obtain a more comprehensive overview of the effects of strong high light on protein expression, all proteins quantified using the non‐targeted approach were statistically validated (anova, Benjamini−Hochberg corrected false discovery rate (FDR): 1%). Out of 3620 quantified proteins, 327 exhibited highly significant changes in abundance upon strong high light treatment (Appendix S3). A heat map generated by hierarchical clustering of significantly altered proteins (Figure 3a) revealed two major clusters representing proteins up‐ and downregulated after 24 h of strong high light exposure. Notably, FNR, LHCSR3, TRX*f1*, and CRX are among those proteins. The subcluster containing TRX*f1* features proteins already highly induced after 4 h (Figure 3b) of strong high light treatment and whose levels remained stable or decreased slightly toward the end of the time course experiment. Half of the total number of other proteins in this cluster was involved in chlorophyll and carotenoid biosynthesis (CTH1B, HEM2, Porphobilinogen deaminase, PPS2, CPX1, CHLI1, MCH1). Moreover, the cluster includes three proteins that operate in the carbon concentrating mechanism (CCP1, LCI9, LCIA). LHCSR3, CRX (both Figure 3c) and FNR (Figure 3d) are located in clusters with more heterogeneous protein compositions. Notably, NTRC1, whose Arabidopsis homolog was implicated in the control of NPQ (Malviya *et al*., 2016), as well as chlorophyll (Perez‐Ruiz *et al*., 2014) and starch biosynthesis (Michalska *et al*., 2009), clusters closely with LHCSR3 (Figure 3c). Two further low CO_2_‐inducible proteins (LCIB, LCIC) are present in the same cluster. Recent studies revealed that LCIB and LCIC interact with each other and presumably form a chloroplast‐localized complex (Mackinder *et al*., 2017). In addition, several proteins related to general or oxidative stress can be observed in the LHCSR3‐CRX and FNR clusters (ClpB, BIP1, PRX4, HSP70B, HSP70C, HSP90B). To link these protein dynamics to photosynthetic performance functional measurements were devised.

### Acclimation to high light reroutes photosynthetic electron transfer

**Figure 4:**
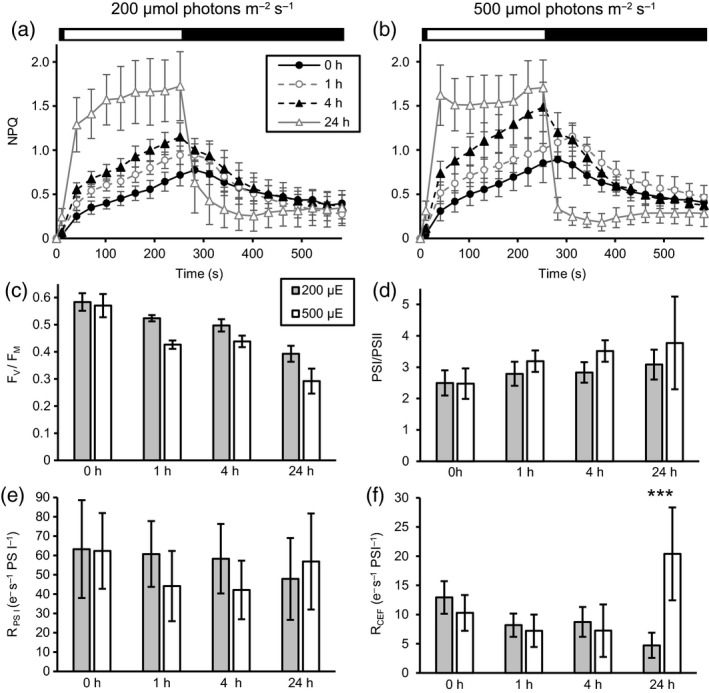
Evolution of photosynthetic parameters in wild type 4A+ cultures upon shift from 20 μmol photons m^−2^ sec^−1^ low light to 200 μmol photons m^−2^ sec^−1^ (200 μE, grey bars) or 500 μmol photons m^−2^ sec^−1^ (500 μE, white bars) high light. Samples were taken from low light grown cultures (0 h) and 1‐, 4‐ and 24 h after the shift to high light. Changes of the nonphotochemical quenching parameter (NPQ; a, b) and of the maximal PSII quantum efficiency (FV/FM, c) were determined by PAM fluorometry. Prior to the measurements, samples were dark incubated for 10 min. NPQ was measured during and after a period of illumination with 800 μmol photons m^−2^ sec^−1^, as indicated by the black‐and‐white bar. Parameters shown in panels (d–f) were determined by absorption spectroscopy. PSI/PSII ratios (d) were calculated from the ratios of the increase in the trans‐thylakoid electric field (ΔΨ) caused by the transfer of one charge across the membrane by either photosystem. ΔΨ caused by the transfer of one charge across the thylakoid membrane by PSI was measured in the presence of PSII inhibitors DCMU (40 μm) and HA (1 mm). Turnover rates of overall photosynthetic electron transfer (RPSI, e) and turnover rates of cyclic electron flow around PSI (RCEF, f) were normalized to the amplitude of one charge transferred across the membrane by PSI. RCEF was measured in the presence of PSII inhibitors DCMU and HA. At 24 h, RCEF is significantly higher in cultures kept under 500 μmol photons m^−2^ sec^−1^ than in cultures kept under 200 μmol photons m^−2^ sec^−1^ (two‐way anova, Bonferroni correction: *P* < 0.001). Values shown represent means of nine independent biological replicates, except for RCEF: five replicates. Results are shown ± standard deviation.

Measurement of NPQ (Figure 4) revealed a clear induction of qE under both light intensities after 24 h (Figure 4a, b). Comparing the development of NPQ under 200 and 500 μmol photons m^−2^ sec^−1^ showed a faster induction under 500 μmol photons m^−2^ sec^−1^. Investigation of the maximal PSII quantum efficiency (Figure 4c), measured as F_v_/F_m_, displayed a more pronounced decrease under 500 μmol photons m^−2^ sec^−1^ as compared with 200 μmol photons m^−2^ sec^−1^. However, the PSI/PSII ratio remained comparable (Figure 4d). In a next step, overall photosynthetic electron transfer rates (R_PSI_) and cyclic electron transfer rates around PSI (R_CEF_) were assessed (Figure 4e,f). R_PSI_ and R_CEF_ were calculated from the initial rates of decay of the transmembrane electric field in the absence (R_PSI_) or presence (R_CEF_) of PSII inhibitors and normalized to the photosystem‐related amplitude of the electric field caused by a saturating, single‐turnover flash in PSII‐inhibited conditions. Under 200 and 500 μmol photons m^−2^ sec^−1^, R_PSI_ did not show significant differences. Analysis of R_CEF_ revealed the largest differences. R_CEF_ decreased under 200 μmol photons m^−2^ sec^−1^ from 0 to 24 h, whereas it was significantly increased under 500 μmol photons m^−2^ sec^−1^ after 24 h. This enhancement of CEF under 500 μmol photons m^−2^ sec^−1^ demonstrated a rerouting of photosynthetic electron transfer in response to high light stress. As phosphorylation of LHCSR3 was induced strongest after 24 h under 200 or 4 h under 500 μmol photons m^−2^ sec^−1^, where R_PSI_ was most affected, an interrelationship between regulation of photosynthetic electron transfer and phosphorylation dynamics of LHCSR3 could be anticipated.

### N‐terminal phosphorylation of LHCSR3 is synergistic and modulated by light

**Figure 5:**
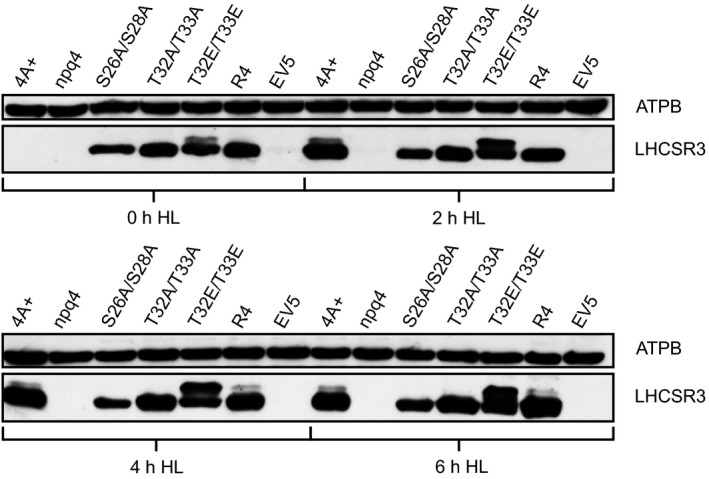
Western blot analysis of whole cell extracts from cultures exposed to 0, 2, 4 or 6 h of 200 μmol photons m^−2 ^sec^−1^ high light. Utilized strains were wild type 4A+, the LHCSR3 knockout strain (npq4), npq4 rescue strains with indicated amino acid alterations in the LHCSR3 protein, expressed under the control of the constitutive PSAD promotor, a rescue strain without amino acid alteration (R4) and an empty vector transformed control strain (EV5). Cultures were pre‐grown heterotrophically at ~20 μmol photons m^−2^ sec^−1^ low light and shifted to autotrophic high light conditions at the timepoint 0 h. For SDS‐PAGE, whole cell samples were adjusted to equal chlorophyll amounts. Immunodetection using an LHCSR3 specific antibody shows changes in abundance of the protein and varying migration behavior of different LHCSR3 species. Immunodetection of the β‐subunit of the chloroplast ATP synthase (ATPB) was used as a loading control.

To evaluate the biological function of the STT7‐dependent LHCSR3 phosphorylation, we altered the N‐terminal phosphorylation sites (S26, S28, T32, T33) of LHCSR3 by site‐directed mutagenesis and nuclear transformation of the LHCSR3‐less *npq4* mutant. Ser residues were changed to Ala, while Thr residues were changed to Ala and Glu. For expression of transgenes, we took advantage of a nuclear expression strategy using the foot‐and‐mouth‐disease virus 2A self‐cleavage peptide to transcriptionally fuse heterologous gene expression to antibiotic resistance in *C. reinhardtii* (Rasala *et al*., 2012). As antibiotic resistance genes we used the *ble* gene (Stevens *et al*., 1996) as described (Rasala *et al*., 2012). Using a PSAD promoter, recombinant LHCSR3 was successfully expressed, even under low light (0 h HL) (Figure 5). Alteration of S26 and S28 as well as T32 and T33 to Ala almost completely abolished phosphorylation of the remaining phosphorylatable residues as the upper LHCSR3 band, attributed to phosphorylated LHCSR3 (see also Figure S3), was missing after SDS‐PAGE separation and immunoblotting (Figure S4). Conversely, replacement of T32 and T33 by Glu triggered a significant increase in phosphorylation of LHCSR3 in comparison with WT (4a+) (Figure 5), suggesting that phosphorylation of T32/33 synergistically supports phosphorylation of S26/28. It is striking that phosphorylation of LHCSR3‐T32E/T33E was already visible under LL conditions in contrast with the LHCSR3 rescued strain (Figure 5). Notably, phosphorylation of WT, of the LHCSR3 rescued strain and particularly of LHCSR3‐T32E/T33E was clearly increasing with prolonged light treatment under 200 μmol photons m^−2^ sec^−1^ (Figure 5), indicating that the phosphorylation was activated by light and therefore confirming results obtained by mass spectrometry (Figures 1, S2).

### Quantitative proteomics indicates that the phosphorylation status of LHCSR3 modulates LHCB4 phosphorylation

**Figure 6:**
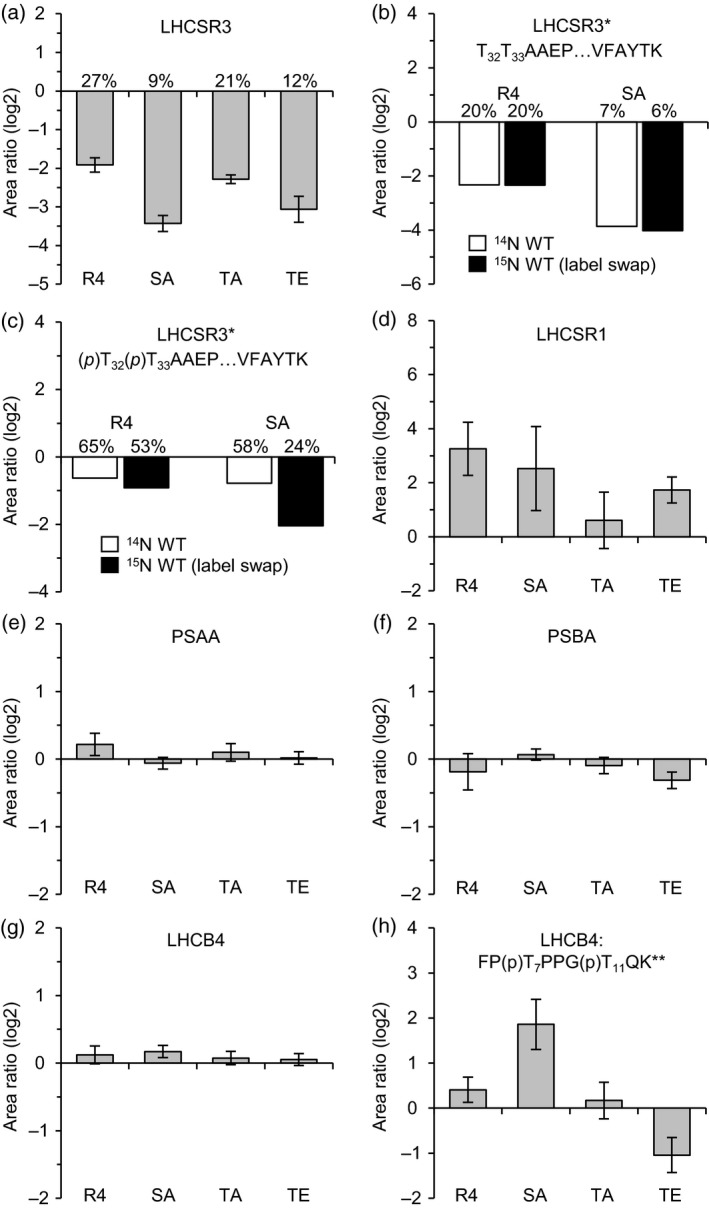
Changes in relative protein/peptide abundances and phosphorylation levels in LHCSR3 mutant and rescued strains. Unlabelled (^14^N) WT and labelled (^15^N) mutant/rescue strains were cultivated in HSM and exposed to high light (200 μmol photons m^−2^ sec^−1^) for 24 h. A replicate experiment was performed with swapped isotopic labels (^15^N WT, ^14^N mutant/rescue). Data were obtained by LC‐MS/MS (PRM) and represent the mean log_2_‐transformed mutant/rescue to WT peptide ratios. A minimum of three peptides was quantified per experiment. Error bars indicate the standard deviation between all peptide ratios of both replicates. Percentages denote expression levels in comparison to the wild type and were derived from mean peptide ratios prior to log2‐transformation. R4, LHCSR3‐R4; SA, LHCSR3‐S26A/S28A; TA, LHCSR3‐T32A/T33A; TE, LHCSR3‐T32E/T33E; *, quantified only in samples treated with trypsin; **, quantified only in samples treated with lysC; (p), phosphorylated residue. (a) LHCSR3. (b, c) Quantification results of the singly phosphorylated (b) and nonphosphorylated (c) versions of the LHCSR3 peptide T_32_T_33_AAEPQTAAPVAAEDVFAYTK. Data from replicate experiments are shown individually. (d) LHCSR1. (e) Photosystem I P700 chlorophyll a apoprotein A1 (PSAA). (f) Photosystem II protein D1 (PSBA). (g) LHCB4. (h) Doubly phosphorylated LHCB4 peptide FP(p)T_7_PPG(p)T_11_QK.

To monitor whether the protein abundance of selected proteins was changed due to the phosphorylation status of LHCSR3, WT, rescued strain and N‐terminal LHCSR3 mutant cells were shifted to 200 μmol photons m^−2^ sec^−1^ for 24 h and subjected to directed quantitative proteome analyses. Total protein extracts from ^15^N labeled WT and one unlabelled ^14^N mutant (LHCSR3‐S26A/S28A, LHCSR3‐T32A/T33A, LHCSR3‐T32E/T33E) or rescued strain were mixed at 1:1 ratio. In addition, a label swap was performed using unlabelled WT and labelled mutants/rescued strain. Samples were split and one aliquot was digested with lysC and one with trypsin. Subsequent LC‐MS/MS experiments performed with samples treated with lysC confirmed the expression of WT and mutant LHCSR3 proteins, as also shown by the immunoblotting experiment (Figure 5), and provided evidence for the phosphorylation of S26 or S28 of all versions of LHCSR3 (peptide: S_26_VS_28_GRR(T/A/E)_32_(T/A/E)_33_AAEPQTAAPVAAEDVFAYTK; Figure S5 and Table S1). Moreover, in tryptically digested samples, a peptide (T_32_T_33_AAEPQTAAPVAAEDVFAYTK) phosphorylated at T32 or T33 was found in both WT and LHCSR3‐S26A/S28A mutant (Figure S5). Determination of peptide abundance ratios (mutant/rescued strain versus WT) via PRM (Figure 6 and Appendix S4) revealed that expression levels of LHCSR3 were diminished in the rescued and mutant strains (expression levels compared with WT: 27% (rescued strain), 9% (LHCSR3‐S26A/S28A), 21% (LHCSR3‐T32A/T33A), 12% (LHCSR3‐T32E/T33E), Figure 6a). The amounts of the nonphosphorylated peptide T_32_T_33_AAEPQTAAPVAAEDVFAYTK were reduced even stronger to average levels of 17% (rescued strain) and 6% (LHCSR3‐S26A/S28A; Figure 6b). In contrast, the average abundance of the singly phosphorylated peptide T_32_T_33_AAEPQTAAPVAAEDVFAYTK was on average reduced to only 51 and 35% in the rescued strain and LHCSR3‐S26A/S28A (Figure 6c). These data point to much higher degrees of Thr phosphorylation in those strains compared with WT. This significant phosphorylation at Thr in the rescued strain and LHCSR3‐S26A/S28A was not detectable in the immunoblot analyses (Figure 5), indicating that Thr phosphorylation alone does not lead to a visible electrophoretic mobility shift after SDS‐PAGE separation, whereas Ser and Thr phosphorylation does. Differences in the phosphorylation status of LHCSR3 in the N‐terminal LHCSR3 mutants were not due to differences in STT7 abundances (Figure S6a and Appendix S4). Only in the LHCSR3 rescued strain, the abundance of STT7 was diminished compared with WT. In all strains, except LHCSR3‐T32A/T33A, LHCSR1 expression was induced (Figure 6d). Amounts of PSI (PSAA (Figure 6e), PSAD (Figure S6b)), PSII (PSBA (Figure 6f)), PSBD (Figure S6c)), the cyt b_6_/f complex (CYT b_6_ (Figure S6d)) and ATPase (ATPA (Figure S6e)) in the rescued strain as well as in the mutant strains were not considerably affected. Also, the minor antenna proteins LHCB4 (Figure 6f) and LHCB5 (Figure S6f) as well as the LHCI antenna protein LHCA3 (Figure S6g) were present in similar amounts. In these analyses, phosphorylation of LHCB4, a heavily phosphorylated protein in the thylakoid membrane of *C. reinhardtii* (Lemeille *et al*., 2010; Bergner *et al*., 2015), could be detected and quantified (Figure 6h). In the respective LHCB4 peptide, concurrent phosphorylation was found at positions T7 and T11. Strikingly, phosphorylation of both LHCB4 Thr residues was significantly diminished by at least factor 2 in LHCSR3‐T32E/T33E. In contrast, in LHCSR3‐S26A/S28A, phosphorylation of LHCB4 was increased more than three‐fold, indicating that phosphorylation of the N‐terminus of LHCSR3 influences phosphorylation of T7 and T11 of LHCB4. In LHCSR3‐T32A/T33A, in contrast with LHCSR3‐T32E/T33E, the N‐terminal LHCB4 phosphorylation was not diminished in LHCB4‐T7 (Figure S6h) and LHCB4‐T7/11 (Figure 6h) but in LHCB4‐T11 (Figure S6i).

### Association of N‐terminal phosphorylated and nonphosphorylated LHCSR3 with photosynthetic multi‐protein complexes

LHCSR3 was found to associate with PSII and PSI (Allorent *et al*., 2013; Tokutsu and Minagawa, 2013; Bergner *et al*., 2015). To analyze whether the N‐terminal phosphorylation of LHCSR3 affects binding of LHCSR3 to PSII and/or PSI, sucrose density gradient fractionation of detergent solubilized thylakoids isolated from cells (WT, LHCSR3 rescued as well as N‐terminal mutant strains) after 24 h under 200 μmol photons m^−2^ sec^−1^ was employed to analyze co‐migration of LHCSR3 with PSII and PSI multi‐protein complexes. Figure S7 shows the corresponding sucrose density gradients after ultracentrifugation. Six green bands could be distinguished. These green bands corresponded, as described before (Bergner *et al*., 2015), to LHCII monomers, LHCII trimers, PSII core complexes, PSI‐LHCI, PSII–LHCII complexes and CEF supercomplexes. Visual inspection of these bands did not show diminishment of PSI‐LHCI and PSII–LHCII complexes in the N‐terminal LHCSR3 mutant strains. To define the amounts of LHCSR3, PSII and PSI in those fractions isolated from WT, LHCSR3 rescued as well as N‐terminal mutant strains, sucrose density gradient fractions were subjected to SDS‐PAGE separation and immunoblotting experiments (Figures S3 and S4). The peaking of PSBA (fractions 8–10) and PSAD (fractions 10–12) (Figures S3a and S4a) revealed the migration of PSII–LHCII and PSI‐LHCI complexes within the sucrose density gradients, respectively. In the sucrose density gradients of WT, LHCSR3 rescued as well as N‐terminal mutant strains LHCSR3‐S26A/S28A and LHCSR3‐T32A/T33A, the LHCSR3 protein co‐migrated with PSBA and PSAD and therefore peaked in PSII as well as in PSI supercomplex fractions (Figure S3a). The amounts of LHCSR3 stemming from LHCSR3‐S26A/S28A were rather low in the PSII and PSI supercomplex fractions, but signals were still clearly visible. The rather weak signal can be explained by the lower expression of LHCSR3 in this strain as compared with WT, LHCSR3 rescued strain and LHCSR3‐T32A/T33A, as seen in the LHCSR3 immunoblotting results after SDS‐PAGE separation of the low sucrose density fractions (16–29; Figure S3b). Expression of LHCSR3 was similarly low in LHCSR3‐T32E/T33E (Figure 6). In this mutant, LHCSR3 peaked with PSII supercomplexes (Figure S4a). In the low sucrose density fractions (16–29; Figure S4a), the LHCSR3 signal was evident but split in a lower and an upper band in the immunoblot analyses, as seen in Figure 5 for this mutant. In an independent experiment, a lower and faint upper band was visible at the position of the PSII supercomplexes (Figure S4a). Also, in this experiment, in the low sucrose density fractions (16–29; Figure S4a), the LHCSR3 signal was evident and split in a lower and an upper band in the immunoblot analyses. Treatment of isolated thylakoid membranes with alkaline phosphatase revealed that in WT, rescued strain and particularly in LHCSR3‐T32E/T33E, the upper LHCSR3 band disappeared, proving that this band represents phosphorylated LHCSR3 protein (Figure S4b). Notably, LHCSR3‐T32A/T33A thylakoids did not show this upper band before alkaline phosphatase treatment, consistent with the fact that mutation of LHCSR3‐T32A/T33A largely abolished LHCSR3 N‐terminal phosphorylation (see also Figure 5). Although lower and upper bands of LHCSR3 in LHCSR3‐T32E/T33E were equally visible after SDS‐PAGE separation and immunoblot analyses of the low sucrose density fractions (16–29; Figure S4a), both bands were diminished from the LHCSR3 immunoblot signal in the PSII and PSI supercomplex fractions. However, as LHCSR3 in LHCSR3‐T32E/T33E possesses a genetically engineered phosphomimic alteration, the presence of the lower band in the PSI supercomplex fraction would indicate binding of LHCSR3 phosphorylated at T32/T33 to PSII–LHCII supercomplexes. Therefore, N‐terminally phosphorylated and nonphosphorylated LHCSR3 associated with photosynthetic multi‐protein complexes, in particular with PSII–LHCII supercomplexes.

To assess the photosynthetic performance of the N‐terminal LHCSR3 mutants in comparison with WT and *npq4*, cells were shifted from Tris‐Acetate‐Phosphate medium (TAP) low light to High‐Salt medium (HSM) high light for 24 h, and qE‐dependent NPQ was measured (Figure S8a). The NPQ was partially rescued in the LHCSR3 rescued strain and LHCSR3‐T32A/T33A, while in LHCSR3‐S26A/S28A and LHCSR3‐T32E/T33E, the rescue was less pronounced. This finding is consistent with the fact that the amounts of LHCSR3 were lower in these two strains (Figure 6). In addition, the PSII quantum yield was assessed from cells shifted from TAP low light to HSM high light for 24 h (Figure S8b). Here, F_v_/F_m_ decreased at high light, while no significant differences were observed among the genetically engineered LHCSR3 transformant strains, supporting the view that none of the recombinant strains was particularly stressed.

### Quantitative proteomics reveals dynamic N‐terminal LHCB4 phosphorylation

**Figure 7:**
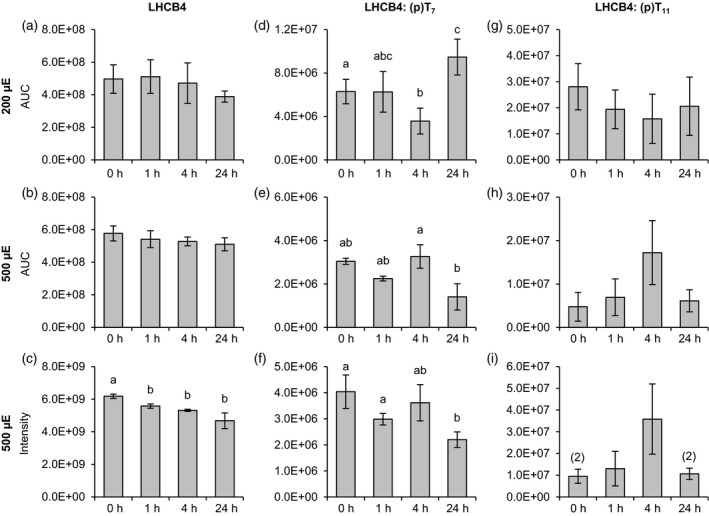
Changes in LHCB4 abundance and phosphorylation (T_7_ and T_11_) in response to different light conditions. Algal cultures were exposed to 200 μmol photons m^−2^ sec^−1^ (a, d, g) or 500 μmol photons m^−2^ sec^−1^ (b, e, h) high light and samples were taken at the indicated time points. Label‐free quantification was performed as described in Figure 1. For the 500 μmol photons m^−2^ sec^−1^ experiment supplementary quantitative data was obtained by MS1 feature detection using the MaxLFQ algorithm (c, f, i). (a−c) Total LHCB4. (d−f) Peptide FP(p)T_7_PPGTQK. (g−i) Peptide FPTPPG(p)T_11_QK. Data represent mean ± standard deviation (200 μmol photons m^−2^ sec^−1^: *n* = 4; 500 μmol photons m^−2^ sec^−1^ (PRM): *n* = 3 (0 h: *n* = 2), 500 μmol photons m^−2^ sec^−1^ (MaxLFQ): *n* = 3). Numbers in parentheses indicate the number of data points if low abundances did not permit quantification in all replicates. Welch's *t*‐test (unpaired, two‐tailed) was used to analyze the data: Values labelled with identical letters, or no letters, do not show statistically significant differences (*P* > 0.05). (p), phosphorylated residue. Only kinetics of protein abundance changes, but not absolute protein or peptide levels are comparable between the two high light experiments, since samples were analyzed with different LC‐MS configurations.

To monitor the phosphorylation status of LHCB4 in the WT in response to light, we performed PRM measurements (Figure 7 and Appendices ;S1 and ;S2) on the same samples used for quantitation of LHCSR3 phosphorylation (Figure 1). LHCB4 amounts were found to be largely stable in the time course experiment (Figure 7a–c). Under 200 μmol photons m^−2^ sec^−1^, the abundances of the N‐terminally phosphorylated peptide LHCB4‐T7 had dipped after 4 h but reached a maximum after 24 h (Figure 6d). LHCB4‐T11 (Figure 7g) showed a similar but less pronounced pattern, although, due to high standard deviations, a clear trend could not be determined for this peptide. Notably, under 500 μmol photons m^−2^ sec^−1^, phosphorylation quantities for LHCB4‐T11 peaked after 4 h and had decreased after 24 h, although changes in abundance were not statistically significant. These patterns were observed in both independent quantitation methods (Figure 7h,i). Conversely, a significant diminishment in phosphorylation amounts after 24 h versus 4 h in the abundances derived from the PRM measurements and versus 1 h in the quantitation results from the non‐targeted approach was also observed for LHCB4‐T7 phosphorylation under 500 μmol photons m^−2^ sec^−1^ (Figure 6e,f).

## Discussion

In this work, we investigated light‐dependent phosphorylation dynamics of LHCSR3 and LHCB4 and analyzed functional consequences of LHCSR3 phosphorylation. Our data revealed that N‐terminal phosphorylation of LHCSR3 impacts LHCB4 N‐terminal phosphorylation. The data also showed that the N‐terminal LHCSR3 multisite phosphorylation is light triggered and synergistic. Interestingly, LHCSR3 phosphorylation is diminished after 24 h under 500 μmol photons m^−2^ sec^−1^, coinciding with a strong onset of CEF.

### N‐terminal phosphorylation of LHCSR3 is a synergistic and dynamic process

It has been shown that LHCSR3 in a mutant deficient in STT7 (*stt7‐1*) is not phosphorylated at its N‐terminus. Nevertheless, it could efficiently bind to putative CEF supercomplexes (Bergner *et al*., 2015). N‐terminal phosphorylation of LHCSR3 amino acid residues T32/33 and S26/28 was absent in LHCSR3‐T32A/T33A and in LHCSR3‐S26A/S28A mutants, respectively, yet LHCSR3 associated with PSII and PSI supercomplexes as observed in WT and LHCSR3 rescued strains. Therefore, phosphorylation is neither required for binding of LHCSR3 to PSII−LHCII nor to PSI−LHCI complexes. In LHCSR3‐T32E/T33E, LHCSR3 phosphorylation was enhanced (Figure 6). The increase in overall phosphorylation of LHCSR3 was likely not due to increased C‐terminal phosphorylation, as phosphorylation of the C‐terminus could not be detected. Accordingly, the increase was likely due to phosphorylation of S26/28 of LHCSR3. Our data confirmed that S26/28 could indeed be phosphorylated when T32/33 were phosphorylated at the same time or when the Thr residues were altered to Ala or Glu (Table S1, Figure S5). The association of doubly (as mimicked by LHCSR3‐T32E/T33E) and triply phosphorylated LHCSR3 with PSII supercomplexes (Figure S4a), although to a small extent, is evident, signifying that phosphorylated LHCSR3 may still associate with PSII supercomplexes. The immunoblot analyses of LHCSR3‐T32E/T33E (Figure 5) also pointed to the fact that mimicking phosphorylation of the Thr residues increased phosphorylation of the S26/28, suggesting that phosphorylation is synergistically controlled. The strong increase of phosphorylation in LHCSR3‐T32E/T33E in the light as well as the absence of S26/28 phosphorylation in *stt7‐1* (Bergner *et al*., 2015) is consistent with STT7 being the responsible kinase, as the redox status of the plastoquinone pool modulates the activity of the STT7 kinase (Vener *et al*., 1997; Zito *et al*., 1999). This is also in line with a recent structural model of LHCSR3, where the N‐terminus of LHCSR3 should be accessible to STT7 from the chloroplast stroma (Ballottari *et al*., 2016). However, only a part of the LHCSR3 pool undergoes double and triple phosphorylation, indicating that the status of LHCSR3 phosphorylation is rather dynamic.

### N‐terminal phosphorylation of LHCSR3 modulates phosphorylation of LHCB4

**Figure 8:**
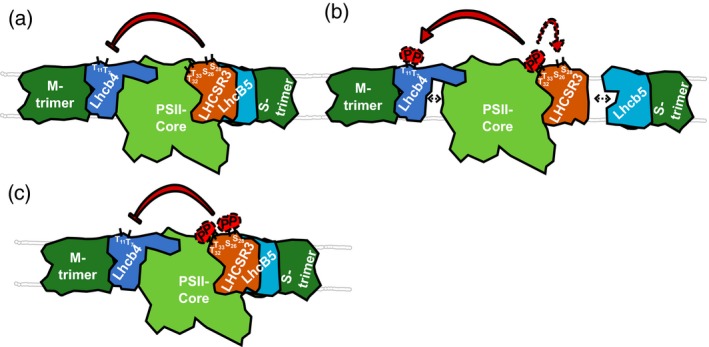
Working model depicting the modulation of LHCSR3 phosphorylation and its influence on LHCB4 phosphorylation and PSII–LHCII complex stability. (a) Presence of LHCSR3 not carrying N‐terminal phosphorylation at Ser 26/28 and/or Thr 32/33 attenuates phosphorylation of LHCB4 threonine residues 7 and 11. (b) Phosphorylation of LHCSR3 Thr 32/33 promotes phosphorylation of Ser 26/28 and of LHCB4 threonine residues 7 and 11. LHCB4 phosphorylation promotes dissociation of the PSII–LHCII supercomplex. (c) Phosphorylation of the N‐terminal LHCSR3 serine and threonine residues suppresses phosphorylation of LHCB4 Thr 7/11.

Our data clearly showed that phosphomimicking of LHCSR3 phosphorylation in LHCSR3‐T32E/T33E decreased the amount of phosphorylation of T7 and 11 of LHCB4 (Figure 6). The transit peptide of LHCB4 in *C. reinhardtii* is not processed and T7 has been found to be phosphorylated before (Turkina *et al*., 2004). In the present study, we confirmed a single and double phosphorylation at positions T7 and T11 (Bergner *et al*., 2015). In a previous study, we showed that S103 of LHCB4 was diminished in phosphorylation in the *stt7‐1* mutant (Bergner *et al*., 2015), indicating that this site is phosphorylated in an STT7‐dependent way. This is in line with the findings that high light‐induced phosphorylations of Lhcb4.1 and Lhcb4.2, occurring at four different sites, are STN7‐dependent in Arabidopsis (Fristedt and Vener, 2011). It is assumed that this STN7‐dependent phosphorylation of Lhcb4.1 and Lhcb4.2 is an important activating impulse for disassembly of the PSII supercomplexes in vascular plants during high light exposure (Fristedt and Vener, 2011). This is particularly important as LHCB4 serves as a linker between PSII core and LHCBM proteins, supporting excitation energy transfer between the trimeric LHCBM proteins and the PSII core (Dainese and Bassi, 1991; Dekker and Boekema, 2005; van Amerongen and Croce, 2013). Moreover, LHCB4 is crucial for state transitions in *C. reinhardtii* (Tokutsu *et al*., 2009), in which phosphorylation of LHCB4, as well as LHCB5, is considered as the driving force for the detachment of LHCBM polypeptides from PSII during the transition from state 1 to state 2. Notably, phosphorylated LHCB4 was not identified bound to PSII core complexes and appeared to be associated with other free LHCBM (Iwai *et al*., 2008). To relate LHCSR3 phosphorylation to LHCB4 phosphorylation, it must be taken into account that, in LHCSR3‐R4, the strain rescued with WT LHCSR3, the amounts of LHCSR3 were diminished to 27% of the WT level; yet, phosphorylation of the LHCB4 phosphosites was increased in this strain. A diminishment in LHCSR3 levels was also true for all other complementation strains. In LHCSR3‐S26A/S28A, LHCB4‐Thr7/11 phosphorylation was significantly increased (Figure 6h). In this strain, T32/33 was phosphorylated and relative phosphorylation levels were about two to three times higher compared with WT. Therefore, in the absence of S26/28 phosphorylation, phosphorylation at T32/33 would increase LHCB4 phosphorylation (Figure 6). In LHCSR3‐T32A/T33A, the N‐terminal LHCB4 phosphorylation was not diminished in LHCB4‐T7 and almost unchanged in LHCB4‐T7/11 and in LHCB4‐T11 (Figures 6h, S6). In contrast, in LHCSR3‐T32E/T33E, which is expected to show a concomitant increase in LHCSR3‐S26 and/or LHCSR3‐S28 phosphorylation, LHCB4‐T7 phosphorylation was not significantly increased as would have been expected by the low LHCSR3 expression level. In this strain, the LHCB4‐T11 and LHCB4‐T7/11 phosphorylations were diminished. Therefore, taking all these observations into account, it appears that: (i) an absence of LHCSR3 likely promotes LHCB4‐T7 and LHCB4‐T7/11 phosphorylation, while its presence suppresses LHCB4‐T7/11 phosphorylation; (ii) the absence of S26/28 phosphorylation and presence of T32/33 phosphorylation alone promotes LHCB4‐T7/11 phosphorylation; and (iii) whereas S26/28 phosphorylation and T32/33 phosphorylation together suppress LHCB4 phosphorylation. This is summarized and depicted in Figure 8. The pronounced impact of the alterations of LHCSR3‐S26/28 and LHCSR3‐T32/33 on LHCB4‐T7/11 phosphorylation implies that LHCSR3 and LHCB4 are in close proximity in the PSII–LHCII supercomplex structure (see also below).

How about the interrelation of LHCSR3 and LHCB4 phosphorylation dynamics in WT under 200 and 500 μmol photons m^−2^ sec^−1^ (Figures 1, 6)? In WT after 24 h under 500 μmol photons m^−2^ sec^−1^, where qE and CEF were strongly induced, LHCB4‐T7 phosphorylation was strongly diminished compared with the 4 h abundances in the PRM quantification or to the 0 and 1 h timepoints in the non‐targeted approach (Figure 7). LHCSR3 phosphorylation under 500 μmol photons m^−2^ sec^−1^ between 4 and 24 h appeared to decrease as well (Figures 1 and S2). Generally, LHCSR3 phosphorylation and LHCB4 phosphorylation follow a similar pattern after the 1 h timepoint under 500 μmol photons m^−2^ sec^−1^ illumination. This observation is corroborated by results from cells kept under 200 μmol photons m^−2^ sec^−1^, where singly T32 or T33 phosphorylated LHCSR3 was strongly increased after 24 h, while doubly phosphorylated LHCSR3 was only detectable at the 4 and 24 h ‐ and triply phosphorylated LHCSR3 only at the 24 h timepoint (Figures 1 and S2), correlating with a significant increase in LHCB4‐T7 phosphorylation (Figure 7). Although the phosphorylation dynamics of individual sites are complex, it appears that overall N‐terminal LHCSR3 phosphorylation at T32/33 promotes LHCB4‐T7/11 phosphorylation. For interpretation of the site‐directed LHCSR3 mutant data, it has to be taken into account that, at peak phosphorylation in WT, LHCSR3 was not entirely phosphorylated at the N‐terminal Thr positions 32/33, while in LHCSR3‐T32E/T33E, the entire LHCSR3 harbors the phosphomimic at T32/33, possibly explaining the strong impact on LHCB4 T7/11 phosphorylation. After 24 h under 500 μmol photons m^−2^ sec^−1^, less LHCSR3 were phosphorylated at the N‐terminal Thr positions 32/33, consistent with a drop in LHCB4‐T7/11 phosphorylation. Therefore, it is tempting to speculate that negative charges at the position of LHCSR3‐T32/33 in the absence of LHCSR3‐S26/28 phosphorylation support N‐terminal LHCB4 phosphorylation, therefore promoting efficient PSII repair.

In the structure of the spinach PSII–LHCII complex at 3.2 Å (Wei *et al*., 2016), three carotenoid and 13 chlorophyll‐binding sites were located to LHCB4, which itself is associated with CP47. The N‐terminal region of LHCB4 forms two motifs with irregular coil structures (motif I and II). Motif II forms a chlorophyll‐binding site that is suggested to serve as interfacial chlorophyll enabling energy transfer between two adjacent antenna complexes (Wei *et al*., 2016), underpinning the importance of the N‐terminal region of LHCB4. Intriguingly, Ido *et al*. identified cross‐linking between PSBP and PSBR as well as PSBP and LHCB5 in PSII‐enriched membranes from spinach using chemical cross‐linking and mass spectrometry (Ido *et al*., 2014). The structure reveals a close interconnection between PSBP and CP43 as well as CP47. According to structural data, PSBP binds in a cleft between the luminal domains of CP43 and CP47 (Wei *et al*., 2016). Unfortunately, the position of PSBR could not be resolved in the spinach PSII–LHCII complex (Wei *et al*., 2016). PSBR conversely is crucial for binding of LHCSR3 to PSII–LHCII complexes (Xue *et al*., 2015). LHCB5 is closely associated with CP43 (Wei *et al*., 2016). In PSII C_2_S_2_ supercomplexes from *C. reinhardtii*, lacking the PSBR subunit, LHCSR3 binds at three different positions including the LHCB5 subunit and the LHCII trimers (Semchonok *et al*., 2017), supporting the presence of LHCSR3 at PSII supercomplexes and its potential to modulate processes in this complex. Moreover, in the presence of LHCSR3, excitation energy transfer from light‐harvesting complexes to CP43 is selectively inhibited in isolated PSII–LHCII supercomplexes (Johar *et al*., 2017), supporting binding of LHCSR3 in the vicinity of LHCB5 and CP43. The impact of LHCSR3 phosphorylation on the N‐terminal phosphorylation of LHCB4 could be indirectly transmitted via PSBP and PSBR, although a direct interconnection of LHCSR3 and LHCB4 cannot be excluded. Interestingly, recent structural data implied that in vascular plants the excitation energy transfer from the furthest antennae to the core likely occurs via LHCB4 and S‐LHCII complexes (Dhote *et al*., 2017). Indeed, structural data on the PSII–LHCII−LHCSR3 supercomplexes obtained via crystallography or high‐resolution cryo‐electron microscopy single particle analysis will be required to pinpoint the binding site(s) of LHCSR3.

### Interplay of LHCSR3 phosphorylation and regulation of electron transfer

Intriguingly, after 24 h under 500 μmol photons m^−2^ sec^−1^, CEF and qE were strongly induced, whereas LHCSR3 phosphorylation overall declined. Therefore, LHCSR3 phosphorylation is not required for efficient qE. It seems rather required for the regulation of LHCB4 phosphorylation, which is connected to PSII–LHCII supercomplex disassembly and promotion of PSII repair, as suggested above. N‐terminal phosphorylation of LHCSR3 is promoted by the redox‐regulated kinase STT7 in *Chlamydomonas* (Lemeille *et al*., 2009; Bergner *et al*., 2015; Singh *et al*., 2016). In Arabidopsis, STN7 is regulated also via protein degradation (Trotta *et al*., 2016), in particular promoted by increasing light intensities and prolonged exposure times. According to our data, STT7 was not strongly degraded after 24 h under 500 μmol photons m^−2^ sec^−1^ in *Chlamydomonas*. Therefore, a change in STT7 activity, as indicated by an alteration of N‐terminal LHCSR3 phosphorylation, is not due to degradation but stems from another mechanism. As the onset of CEF is redox dependent with an apparent midpoint potential of −306 mV (Strand *et al*., 2016), it is tempting to speculate that STT7 itself is redox inactivated at a similar redox potential due to reduction of a luminal disulfide as proposed (Lemeille *et al*., 2009). In this scenario, CEF is activated, while LHCSR3 phosphorylation declines. The strong induction of CEF and qE under 500 μmol photons m^−2^ sec^−1^ after 24 h (Figure 4) is accompanied with a significant induction of LHCSR3, CRX and FNR expression (Figures 1a–c, 2g–l, 3 and S1), and an induction of CCM‐related proteins (Figure 3). In particular, CCM requires additional ATP (Lucker and Kramer, 2013), in line with the induction of CEF. In vascular plants, FNR is considered to contribute to the partitioning of photosynthetic electron transport (Joliot and Johnson, 2011). Association of FNR with PSI would therefore promote LEF, while CEF might operate via association of FNR with the cyt *b*_*6*_ */f* complex (Zhang *et al*., 2001). In *C. reinhardtii*, binding of FNR to a CEF supercomplex, containing PSI and cyt *b*_*6*_ */f* complexes, was demonstrated in state 2 conditions (Iwai *et al*., 2010) and anoxia (Terashima *et al*., 2012; Mosebach *et al*., 2017). Moreover, it was found that FNR bound to PSI is competent in light‐driven electron transfer from PSI to NADP^+^ via soluble FDX1 (Takahashi *et al*., 2014; Mosebach *et al*., 2017). However, it is unclear whether the PSI‐bound FNR is also functionally involved in CEF. The strong induction of FNR under 500 μmol photons m^−2^ sec^−1^ where CEF is stimulated implies a possible involvement in CEF. The strong increase in CRX protein expression suggests oxidative stress (Hochmal *et al*., 2016), which is also underpinned by the strong expression of LHCSR3 enabling efficient qE. Therefore, under these prolonged high light conditions, up‐regulation of CEF, qE and ROS detoxification work together to defend against oxidative damage. At the same time, down‐regulation of LHCSR3 phosphorylation would contribute to PSII stability; therefore assure efficient qE via bound LHCSR3. Therefore, this system is highly balanced between oxidative stress avoidance and photosynthetic performance.

## Experimental Procedures

### Strains and growth conditions

The *C. reinhardtii* wild type 4a+ was obtained from J.‐D. Rochaix (University of Geneva). LHCSR3 phosphosite mutants (N‐terminus: S26A/S28A; T32A/T33A, T32E/T33E) and LHCSR3 rescued strains were obtained by site‐directed mutagenesis and subsequent transformation into the LHCSR3 knockout background (*npq4*; (Peers *et al*., 2009)). Overexpressing strains were isolated based on the zeocin resistance conferred by the transformation vector. Expression of LHCSR3 with substituted N‐terminal residues was controlled by the PSAD promotor. Strains were maintained on TAP medium solidified with 1.5% w/v agar at 25°C in the presence of ~50 μmol photons m^−2^ sec^−1^ photosynthetically active, continuous illumination. Phosphosite mutants and rescued strains were maintained in the presence of 10 μg ml^−1^ zeocin. For experiments, strains were cultured in TAP medium on a rotary shaker (120 rpm) at 25°C in the presence of ~20 μmol photons m^−2^ sec^−1^ photosynthetically active, continuous illumination. Phosphosite mutants and rescued strains were cultured in the presence of 2 μg ml^−1^ zeocin. 4a+, LHCSR3 phosphosite mutants and the LHCSR3 rescued strain were isotopically labelled with ^15^N/^14^N as described in (Hohner *et al*., 2013). For high light experiments cultures were directly shifted to ~200 μmol photons m^−2^ sec^−1^ upon the shift to HS medium with chlorophyll concentrations set to 4 μg ml^−1^. During the experiment, phosphosite mutants and rescued strains were cultivated in the presence of 0.1 μg ml^−1^ zeocin.

For the 200 μmol photons m^−2^ sec^−1^ versus 500 μmol photonsm^−2^ sec^−1^ kinetics, cultures were maintained in ~20 μmol photons m^−2^ sec^−1^ light in HS medium for 16 h before shifting them to the respective light intensities. Samples were taken 0, 1, 4 and 24 h after the shift.

### Isolation of thylakoid membranes

Thylakoid membranes where isolated from whole cells according to published procedures (Chua and Bennoun, 1975).

### Thylakoid solubilization

For the analysis of membrane associated complexes, isolated thylakoid membranes were diluted to chlorophyll concentrations of 0.4 mg ml^−1^ and solubilized with 1% (w/v) n‐dodecyl‐α‐d‐maltoside for 5 min in darkness at room temperature.

### Sucrose density gradient ultracentrifugation and PSI purification

For analysis of thylakoid membrane complexes, separation of solubilized thylakoids on a 1.3 to 0.1 m sucrose density gradient by ultracentrifugation was performed as described previously (Tokutsu *et al*., 2012).

### SDS‐PAGE, western blot and ECL detection

Samples were adjusted to equal chlorophyll (whole cells (Figure 5) and thylakoids (Figure S4)), equal protein (Figure S1) or equal volume (sucrose density gradient fractions), supplemented with SDS‐PAGE loading buffer and incubated at 65°C for 17 min. Proteins were separated by 13% (w/v) SDS‐PAGE (Laemmli, 1970), blotted onto nitrocellulose membranes (Amersham) and detected by specific antibodies: LHCSR3 (Naumann *et al*., 2007), PSBA (Agrisera), PSAD (Naumann *et al*., 2005), ATPB (Agrisera), CRX (Hochmal *et al*., 2016), FNR (kind gift of F.A. Wollman, IBPC Paris) and CYTF (Agrisera).

### Thylakoid dephosphorylation assay

Isolated thylakoids were incubated with 2 Units CIAP (Promega, http://www.promega.com) per 3 μg chlorophyll for 1 h at 28°C (6 μg total chlorophyll, 50 μl total volume).

### Chlorophyll fluorescence measurements

Chlorophyll fluorescence was measured using a Maxi‐Imaging Pulse‐Amplitude‐Modulation (PAM) chlorophyll fluorometer. For each sample three technical replicates were performed. Samples were dark incubated for at least 10 min prior to each measurement. NPQ induction was assessed via a protocol applying photosynthetically active radiation with an intensity of 800 μmol photons m^−2^ sec^−1^ for 3.5 min and following the subsequent relaxation in the dark for 5.5 min. NPQ was calculated as (F_max_ − F_max_’)/F_max_’, where F_max_ and F_max_^’^ represent the maximum fluorescence emission in the dark‐adapted or the light‐adapted state, respectively. F_V_/F_M_ was calculated as (F_max_ − F_0_)/F_max_, where F_0_ is the fluorescence of the sample in the dark‐adapted state.

### Absorption spectroscopy

Time‐resolved absorption changes were measured using a JTS10 spectrophotometer (BioLogic), equipped with a dye laser emitting at 640 nm, pumped by the second harmonic of a Minilite II Nd:YAG laser (Continuum). All *in vivo* spectroscopy was performed using the JTS10. For these measurements, cells were harvested and resuspended to a chlorophyll concentration of 20 μg ml^−1^ in a HEPES−KOH buffer adjusted to pH 7.2, containing 10% (w/v) Ficoll to avoid cell sedimentation. Before measurements, samples were dark incubated for at least 20 min on a shaker set to ~120 rpm.

*In vivo* absorption spectroscopy methods took advantage of the possibility to measure changes in the trans‐thylakoid electric field (ΔΨ) in response to photosynthetic activity directly as absorption changes of pigments embedded in the thylakoid membrane (the ‘electrochromic shift’, see (Bailleul *et al*., 2010) for a review), detected at 520 nm and corrected with signals measured at 546 nm for non‐ΔΨ specific contributions.

PSI/PSII ratio was determined as described previously (Bailleul *et al*., 2010) by evaluating the amplitude of the photosystem associated initial phase of the increase of ΔΨ following a saturating single‐turnover flash in the presence of the photosystem II inhibitors 3‐(3,4‐dichlorophenyl)‐1,1‐dimethylurea (DCMU; 40 μm) and hydroxylamine (HA; 1 mm), yielding an amplitude corresponding to the relative amount of active PSI, or in the absence of inhibitors yielding an amplitude corresponding to the relative amount of active PSI and PSII together.

Cyclic electron transfer rates (R_CEF_) and overall electron transfer rates (R_PSI_) were determined similar to the method described in Joliot and Joliot (2002). To measure in steady‐state conditions, samples were pre‐illuminated for 6 sec with ~300 μmol photons m^−2^ sec^−1^ of actinic light, with the irradiation wavelength peaking around 630 nm. For R_CEF_, cells were poisoned with 40 μm DCMU and 1 mm HA to inhibit PSII, while for R_PSI_ no inhibitors were added. Rates were computed from the initial (3‐ to 5 msec) phase of decay of ΔΨ upon switching off illumination, normalized to the amplitude of ΔΨ change for one electron transferred across the membrane per PSI taken from the ΔΨ increase upon a saturating single‐turnover flash in the presence of PSII inhibitors, allowing expression in e^−^ PSI^−1^ sec^−1^.

### Sample preparation for mass spectrometry

#### LHCSR3 phosphosite mutants

Each unlabeled mutant or the rescue strain was mixed with the ^15^N‐labelled WT strain based on equal amounts of chlorophyll. A label swap experiment was performed with labelled mutant/rescue strains and unlabeled WT. Cells were lysed immediately upon mixing in 4% SDS in 100 mm Tris−HCl (pH 8) in the presence of protease and phosphatase inhibitors (1 mm benzamidine, 1 mm PMSF, 10 mm sodium fluoride, 1 mm sodium orthovanadate, 10 mm sodium pyrophosphate and 10 mm β‐glycerophosphate). Protein concentrations were determined using the Pierce BCA Protein Assay Kit (Thermo Fisher Scientific, http://www.thermofisher.com) according to the manufacturer's instructions. Samples were split into two aliquots, each containing 75 μg of protein, and digested using trypsin and lysC (enzymes to protein ratio 1:50, both enzymes obtained from Promega), respectively, in 0.5 ml ultracentrifugation devices (Amicon Ultra 0.5, 30 kDa cutoff) according to the FASP protocol (Wiśniewski *et al*., 2009). After overnight digestion at 37°C, a fraction of each peptide sample corresponding to 50 μg of protein was submitted to phosphopeptide enrichment using titanium dioxide tips (NT3TIO, Glygen) according to the manufacturer's instructions. Residual peptide solutions were dried by vacuum centrifugation and stored at −80°C (‘nonenriched samples’). LC‐MS/MS analyses of phosphopeptide‐enriched samples were performed exclusively by DDA, since they were required only for the construction of spectral libraries. All LC‐MS/MS‐based quantifications were carried out using nonenriched peptide samples.

### High light kinetics (200 μmol photons m^−2^ sec^−1^ versus 500 μmol photons m^−2^ sec^−1^)

For protein quantitation, a label‐free approach was chosen. Cells were harvested by centrifugation for 5 min at 1500 ***g***. Supernatants were removed and cell pellets were immediately frozen in liquid nitrogen. Cell lysis, protein isolation and determination of protein concentrations were performed as described above. Proteins (2 mg per sample) were on‐filter digested (FASP) in 15 ml ultracentrifugation devices (Amicon Ultra 15, 30 kDa cutoff) using trypsin. Peptides were desalted with Sep‐PAK tC18 cartridges (Waters) and eluted in 2 ml 50% acetonitrile, 0.1% trifluoroacetic acid. A sample volume corresponding to 25 μg of peptides was dried by vacuum centrifugation and stored at −80°C (‘nonenriched samples’). The other fraction was directly subjected to phosphopeptide enrichment by SIMAC (sequential elution from immobilized metal ion affinity chromatography, (Thingholm *et al*., 2008)). Phosphopeptide‐enriched samples were desalted using Pierce™ Graphite Spin Columns according to the manufacturer's instructions (Thermo Fisher Scientific), dried by vacuum centrifugation and stored as described above. Like in the LHCSR3 phosphosite mutant experiments, phosphopeptide‐enriched samples were subjected to DDA analyses only.

### Mass spectrometry and data processing

All LC‐MS/MS analyses were performed on a system consisting of an Ultimate 3000 nanoLC (Thermo Scientific) coupled via a nanospray interface to a Q Exactive Plus mass spectrometer (Thermo Scientific).

### Mass spectra acquisition by DDA for construction of spectral libraries

For details on LC‐MS/MS parameters see Table S2. All enriched and several nonenriched samples were analyzed employing a ‘Top12’ method consisting of a survey scan (MS1) followed by fragmentation of the 12 most abundant ions by higher energy C‐trap dissociation (MS2).

Spectra files were searched using MaxQuant (version 1.5.5.1; Cox and Mann, 2008)) against the *Chlamydomonas* JGI 5.5 protein database which was merged with the translated *C. reinhardtii* NCBI databases of the complete chloroplast (BK000554.2) and mitochondrial (NC_001638.1) genomes. Furthermore, the function ‘include contaminants’ was activated in MaxQuant. Carbamidomethylation of cysteines was set as fixed modification. Variable modifications allowed were oxidation of methionines, acetylation of protein N‐termini and phosphorylation of serine, threonine and tyrosine. Peptide and protein identifications were filtered to satisfy a false discovery rate of 1%. MaxQuant results were imported into Skyline (version 3.6; MacLean *et al*., 2010) for the generation of spectral libraries and inclusion lists, which were required for subsequent targeted peptide quantification by PRM. As MaxQuant does not support identification of ^15^N labelled peptides, only spectra derived from 32n labeled peptides contributed to the spectral libraries. In addition, libraries created from numerous LC‐MS/MS analyses performed in our lab in recent years were used.

### Targeted protein and peptide quantification by PRM

Inclusion lists compiled with Skyline were used for scheduled fragmentation of target peptides. Peptide quantifications were performed using nonenriched samples only. See Table S3 for details on LC and MS parameters.

#### LHCSR3 phosphosite mutants

Raw PRM spectra files were imported into Skyline for the extraction of ion chromatograms and integration of fragment ion peak areas. The total peak areas (area under curve, AUC) of a minimum of three fragment ions per peptide were determined with manual adjustment of peak borders. Data points were discarded if the correlation score (dotp) between the measured product ion peak areas and the fragment ion intensities in the spectral library was lower than 0.7. Then protein ratios (mutant/rescue versus wild type) were calculated using the mean ^14^N/^15^N (label swap: ^15^N/^14^N) ratios of at least three distinct proteotypic peptides per protein after combining Skyline results from the lysC and trypsin treated samples. In case a peptide was quantified in lysC and trypsin‐digested samples, the mean ratio was used for further calculations. The median of all ^14^N/^15^N peptide ratios of a sample was used for normalization to account for small inaccuracies that occurred during mixing of labelled and unlabeled protein prior to digestion. Standard deviations were calculated using ratios of all quantified peptides of a protein.

#### High light kinetics (200 μmol photons m^−2^ sec^−1^ versus 500 μmol photons m^−2^ sec^−1^)

Raw MS data were imported as described above. The integrated areas (AUC) of at least three fragment ions were summed to determine peptide abundances. Data points were discarded if the correlation score (dotp) between the measured product ion peak areas and the fragment ion intensities in the spectral library was lower than 0.7. Total protein abundances were expressed as the sums of the AUCs of a minimum of two peptides. Data were normalized to the total AUC of three peptides from chloroplast GAP‐DH (GAP3, Cre01.g010900). Standard deviations were calculated over all replicates (200 μmol photons m^−2^ sec^−1^: *n* = 4, 500 μmol photons m^−2^ sec^−1^: *n* = 3).

### Non‐targeted protein quantification (500 μmol photons m^−2^ sec^−1^)

All peptide samples from the 500 μmol photons m^−2^ sec^−1^ experiment (three replicates per timepoint) were analyzed by LC‐MS/MS using the DDA scheme described above for the construction of spectral libraries. Spectra files were searched in MaxQuant (version 1.5.8.3) against the aforementioned protein sequence databases. Carbamidomethylation of cysteine was considered as fixed modification. Oxidation of methionine, acetylation of the protein N‐terminus, and phosphorylation of serine, threonine and tyrosine were set as variable modifications. Precursor mass tolerance was set to 10 and 4.5 ppm for first and main search, respectively. Fragment ion tolerance was 20 ppm. The maximum number of missed cleavages (trypsin) allowed was two, minimum peptide length was seven amino acids, and maximum peptide mass was 5500 Da. The feature ‘match between runs’ was activated with the following settings: 0.7 min match time window and 20 min alignment time window. Peptide and protein identifications were filtered to satisfy a false discovery rate of 1%. Label‐free quantification (LFQ) was performed using the MaxLFQ algorithm implemented in MaxQuant. The LFQ minimum ratio count was 2 and only unique peptides were considered for quantification.

Protein lists including LFQ intensities were imported into Perseus (version 1.6.0.2; Tyanova *et al*., 2016). Common contaminants were filtered out, followed by log_2_ transformation of LFQ intensities. Proteins with less than 9 LFQ intensity values across all samples were removed. Remaining missing values were imputed from a normal distribution using default parameters. Multiple sample testing (anova) was performed for all proteins across all timepoints (3 biological replicates each). The false discovery rate was set to 1% using the Benjamini−Hochberg method. After z‐scoring of log_2_ transformed LFQ intensities, proteins were subjected to hierarchical clustering (average linkage, Euclidean distance).

## Conflict of interest

The authors declare no conflict of interest.

## Author contributions

MH and MS designed the research. MS, HX, PG, LM and SVB performed experiments. MS, HX, PG, LM, SVB and MH analyzed data. MH wrote the paper with the help of PG, LM and MS.

## Supporting information


**Figure S1.** Western blot analysis of whole cell extracts from cultures exposed to 200 μmol photons m^−2^ sec^−1^ or 500 μmol photons m^−2^ sec^−1^ high light.

**Figure S2.** Changes in the abundance of singly and multiply phosphorylated variants of the LHCSR3 peptide S_26_VS_28_GRRT_32_T_33_AAEPQTAAPVAAEDVFAYTK in response to different light conditions.

**Figure S3.** Immunoblot analysis of solubilized LHCSR3‐T32E/T33E thylakoids separated by sucrose density gradient centrifugation and analysis of phosphorylation dependent running behavior of LHCSR3 in SDS‐PAGE.

**Figure S4.** Immunoblot analysis of the distribution of PSII, PSI and LHCSR3 across sucrose density gradients.

**Figure S5.** Annotated fragmentation spectra of nonphosphorylated and phosphorylated versions of the N‐terminal LHCSR3 peptide (S/A)V(S/A)GRR(T/A/E)(T/A/E)AAEPQTAAPVAAEDVFAYTKSA.

**Figure S6.** Changes in relative protein/peptide abundances and phosphorylation levels in LHCSR3 mutant and rescued strains.

**Figure S7.** Analysis of the thylakoid membrane composition in strains expressing LHCSR3 with altered N‐terminal phosphorylation sites by sucrose density gradient separation.

**Figure S8.** qE‐dependent NPQ and maximum quantum yield in WT, S26A/S28A, T32A/T33A, T32E/T33E, R10 (equivalent to R4) and npq4.

**Table S1.** Observed phosphopeptides of LHCSR3 (wild type and mutant versions).

**Table S2.** LC‐MS/MS parameters for DDA analyses (for generation of spectral libraries and non‐targeted quantification by MS1 feature detection).

**Table S3.** LC‐MS/MS parameters for PRM analyses.

**Appendix S1.** Raw PRM quantification data of the 200 μmol photons m^−2^ sec^−1^ high light experiment.

**Appendix S2.** Raw PRM quantification data of the 500 μmol photons m^−2^ sec^−1^ high light experiment.

**Appendix S3.** Raw PRM quantification data of the LHCSR3 phosphosite mutants.

**Appendix S4.** Quantification data (non‐targeted/LFQ) of the 500 μmol photons m^−2^ sec^−1^ high light experiment.

## Data Availability

The mass spectrometry proteomics data (DDA) have been deposited to the ProteomeXchange Consortium (http://proteomecentral.proteomexchange.org) via the PRIDE partner repository (Vizcaíno *et al*., 2016) with the dataset identifier PXD010160. All Skyline data sets (PRM) have been uploaded to PanoramaWeb (Sharma *et al*., 2014) and are available under the following web address: https://panoramaweb.org/o22sGD.url.
