# Supplementary material for: Light‐dependent N‐terminal phosphorylation of LHCSR3 and LHCB4 are interlinked in Chlamydomonas reinhardtii
Source: Plant J. 2019 May 30;99(5):877–94. doi: 10.1111/tpj.14368 (PMC6851877; doi:10.1111/tpj.14368)
Supplement: Supplementary file 1 — Figure S1. Western blot analysis of whole cell extracts from cultures exposed to 200 μmol photons m−2 sec−1 or 500 μmol photons m−2 sec−1 high light. [file TPJ-99-877-s001.pdf]

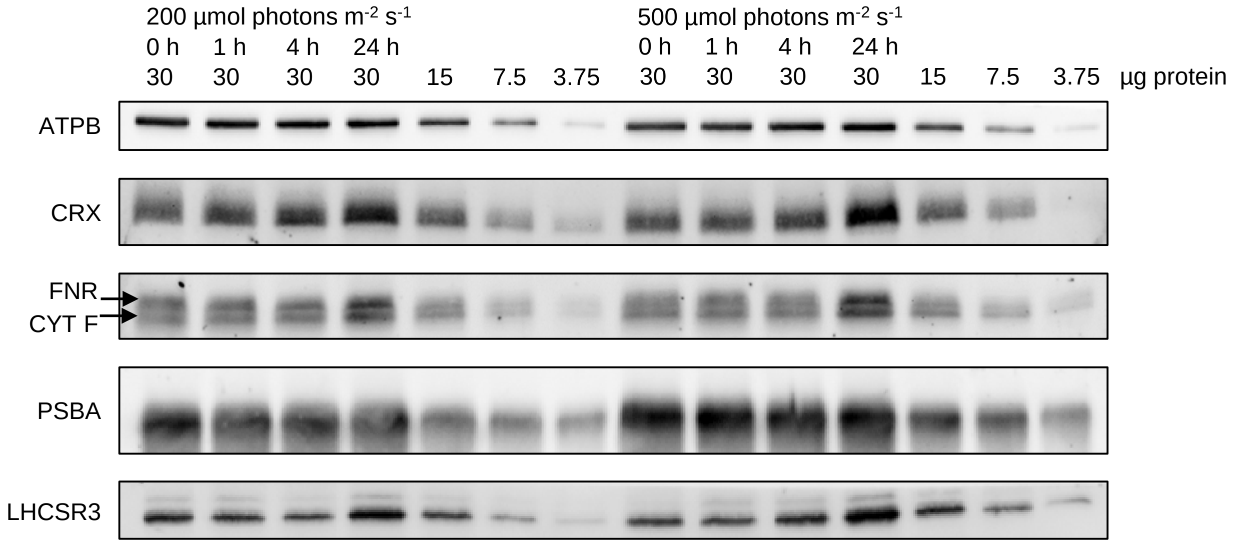

Figure S1. Western Blot analysis of whole cell extracts from cultures exposed to 200 µmol photons m^{-2} s^{-1} or 500 µmol photons m^{-2} s^{-1} high light. Samples were taken after 0 h, 1 h, 4 h and 24 h. For SDS-PAGE, whole cell samples were adjusted to equal protein amounts.
