# Supplementary material for: Light‐dependent N‐terminal phosphorylation of LHCSR3 and LHCB4 are interlinked in Chlamydomonas reinhardtii
Source: Plant J. 2019 May 30;99(5):877–94. doi: 10.1111/tpj.14368 (PMC6851877; doi:10.1111/tpj.14368)
Supplement: Supplementary file 2 — Figure S2. Changes in the abundance of singly and multiply phosphorylated variants of the LHCSR3 peptide S26VS28GRRT32T33AAEPQTAAPVAAEDVFAYTK in response to different light conditions. [file TPJ-99-877-s002.pdf]

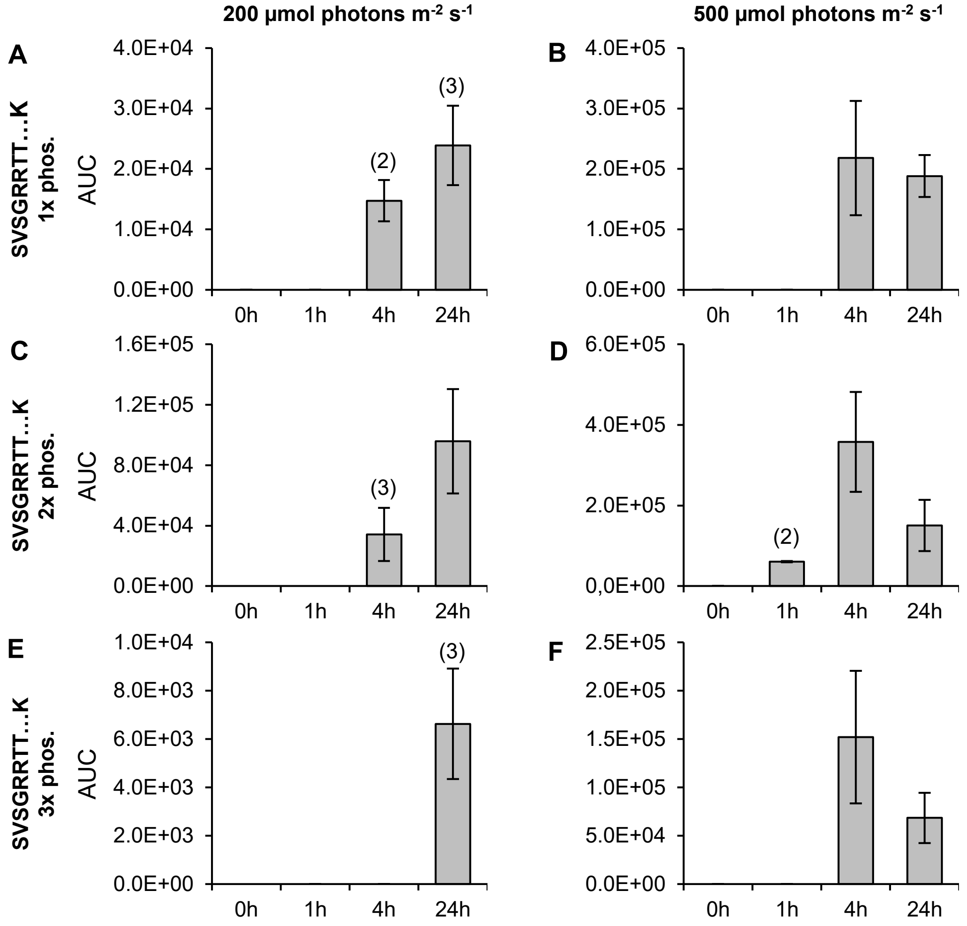

Figure S2. Changes in the abundance of singly and multiply phosphorylated variants of the LHCSR3 peptide $S_{26}VS_{28}GRRT_{32}T_{33}$AAEPQTAAPVAAEDVFAYTK in response to different light conditions.

Algal cultures were exposed to 200 µmol photons m^{-2} s^{-1} (A, C, E) or 500 µmol photons m^{-2} s^{-1} (B, D, F) high light in HS medium and samples were taken at the indicated timepoints. Label-free quantification was performed as described in Figure 1.
(A, B) Singly phosphorylated peptide $S_{26}VS_{28}GRRT_{32}T_{33}$AAEPQTAAPVAAEDVFAYTK
(C, D) Same peptide as in (A,B), doubly phosphorylated.
(E, F) Same peptide as in (A,B), triply phosphorylated.
Data represent mean +/- standard deviation (200 µmol photons m^{-2} s^{-1} : *n*=4; 500 µmol photons m^{-2} s^{-1}: *n*=3 (0h: n=2)). Numbers in parentheses indicate the number of data points if low abundances did not permit quantification in all replicates. Welch's t-test (unpaired, two-tailed) was used to analyse the data: Values labelled with identical letters, or no letters, don't show statistically significant differences ($p > 0.05$).
Only kinetics of protein abundance changes but not absolute protein or peptide levels are comparable between the two high light experiments, since samples were analysed with different LC-MS configurations.
