# Supplementary material for: Light‐dependent N‐terminal phosphorylation of LHCSR3 and LHCB4 are interlinked in Chlamydomonas reinhardtii
Source: Plant J. 2019 May 30;99(5):877–94. doi: 10.1111/tpj.14368 (PMC6851877; doi:10.1111/tpj.14368)
Supplement: Supplementary file 3 — Figure S3. Immunoblot analysis of solubilized LHCSR3‐T32E/T33E thylakoids separated by sucrose density gradient centrifugation and analysis of phosphorylation dependent running behavior of LHCSR3 in SDS‐PAGE. [file TPJ-99-877-s003.pdf]

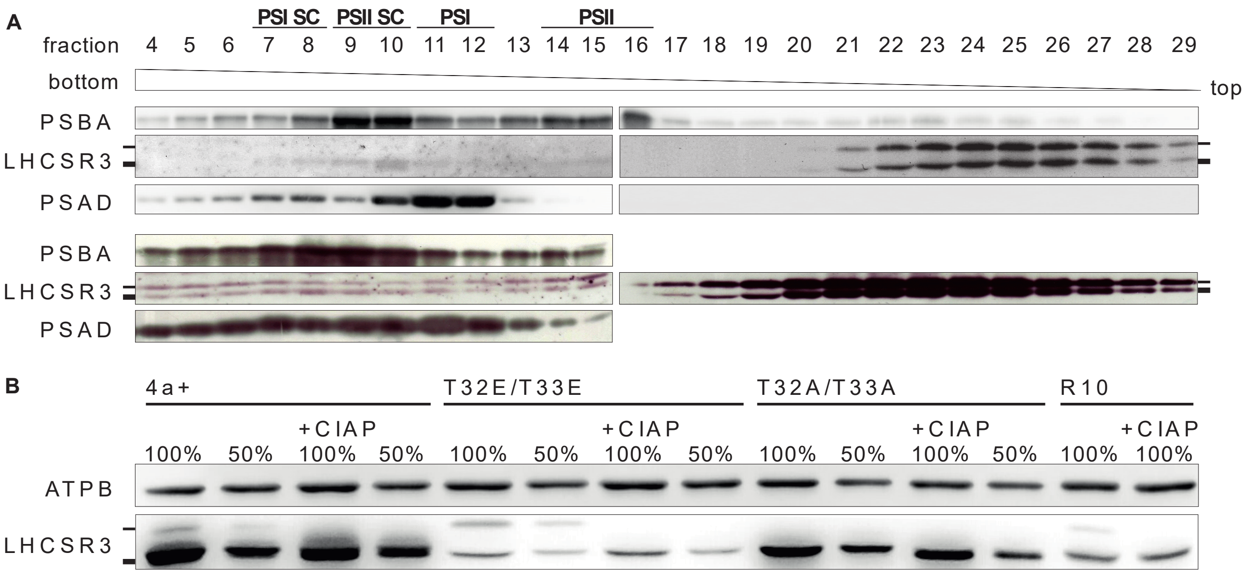

Figure S3. Immunoblot analysis of solubilized LHCSR3-T32E/T33E thylakoids separated by sucrose density gradient centrifugation and analysis of phosphorylation dependent running behavior of LHCSR3 in SDS-PAGE.

(A) Abundance of PSBA, LHCSR3 and PSAD in sucrose density gradient (SDG) fractions obtained from a strain expressing LHCSR3-T32E/T33E. Thylakoids were isolated after 24 h at 200 µmol photons m^{-2} s^{-1}. Prior to the high light exposure, cultures were shifted to autotrophic medium (HSM) and adjusted to 4 µg mL^{-1} chlorophyll (chl) concentration. Isolated thylakoids were solubilized with 1% n-dodecyl-α-D-maltoside followed by SDG centrifugation. Samples for immunoblots were adjusted to equal volume (100 µL of each SDG fraction). The figure shows two independent experiments. The lower, bold black bar indicates the position of the 25 kDa band of the molecular weight marker. The upper black bar indicates the running height of the upper LHCSR3 band.
(B) Abundance of LHCSR3 in thylakoids treated with calf intestinal alkaline phosphatase (CIAP). Thylakoids were isolated from 4A+ and strains expressing LHCSR3-T32E/T33E, -T32A/T33A or wild type LHCSR3 (R10, equivalent to strain R4) under the constitutive PSAD promotor, after 24 h at 200 µmol photons m^{-2} s^{-1}. Prior to the high light exposure, cultures were shifted HSM and adjusted to 4 µg mL^{-1} chl concentration. Isolated thylakoids were adjusted to equal chl concentration (120 µg mL^{-1}) and treated with CIAP asindicated. 3 µg chl (100%) and 1.5 µg chl (50%) were used for SDS-PAGE and Western Blots. ATPB was used as a loading control. The bold, lower black bar indicates the position of the 25 kDa band of the molecular weight marker. The upper black bar indicates the running height of the upper LHCSR3 band.
