# Supplementary material for: Light‐dependent N‐terminal phosphorylation of LHCSR3 and LHCB4 are interlinked in Chlamydomonas reinhardtii
Source: Plant J. 2019 May 30;99(5):877–94. doi: 10.1111/tpj.14368 (PMC6851877; doi:10.1111/tpj.14368)
Supplement: Supplementary file 4 — Figure S4. Immunoblot analysis of the distribution of PSII, PSI and LHCSR3 across sucrose density gradients. [file TPJ-99-877-s004.pdf]

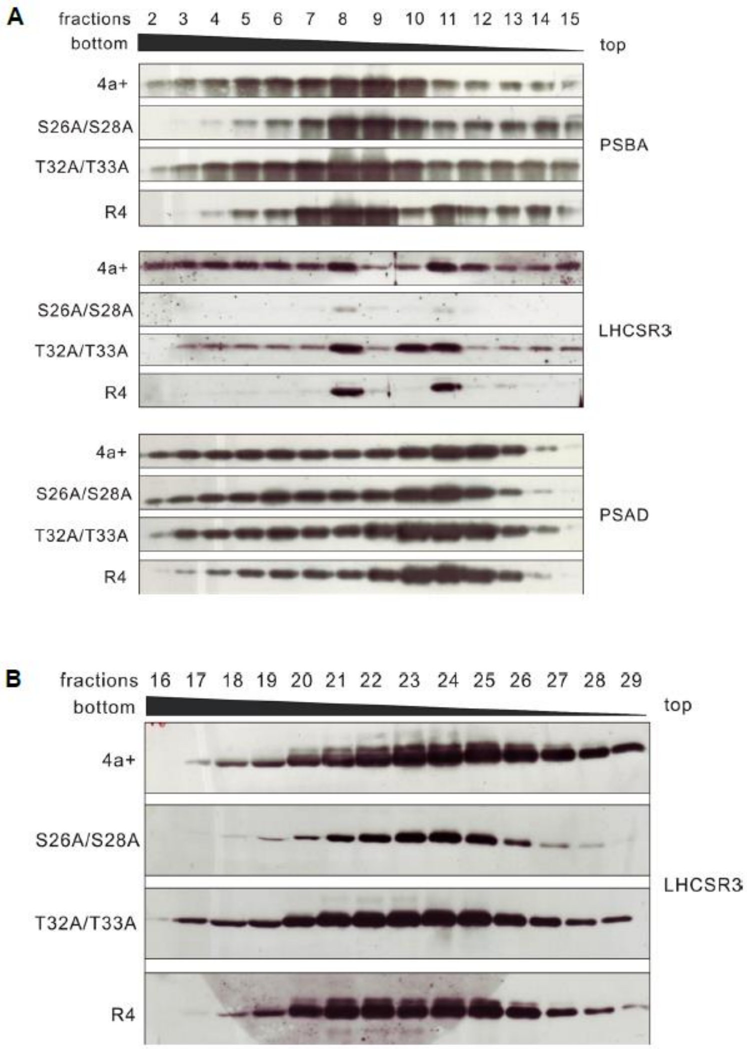

Figure S4. Immunoblot analysis of the distribution of PSII, PSI and LHCSR3 across sucrose density gradients.

Thylakoid membranes were isolated from isolated from wild type 4a+ and strains expressing LHCSR3 with altered N-terminal phosphosites (S26A/S28A, T32A/T33A) or wild typical LHCSR3 (R4) under the control of the constitutively active PSAD promotor. Prior to the thylakoid isolation, strains were grown in autotrophic conditions at 200 µmol photons m^{-2} s^{-1} for 24 h. After solubilization by 1 % n-dodecyl-α-D-maltoside and separation on sucrose density gradients, gradients were separated into 29 fractions, beginning with high sucrose density fractions at the bottom. Fractions were further analyzed by SDS-PAGE and Immunoblot. Samples for SDS-PAGE were adjusted to the equal volume.

(A) Fractions 2-15, analyzed using antibodies targeting PSII subunit PSBA, PSI subunit PSAD and LHCSR3.

(B) Fractions 16-29 analyzed using antibodies targeting LHCSR3.
