# Supplementary material for: Light‐dependent N‐terminal phosphorylation of LHCSR3 and LHCB4 are interlinked in Chlamydomonas reinhardtii
Source: Plant J. 2019 May 30;99(5):877–94. doi: 10.1111/tpj.14368 (PMC6851877; doi:10.1111/tpj.14368)
Supplement: Supplementary file 5 — Figure S5. Annotated fragmentation spectra of nonphosphorylated and phosphorylated versions of the N‐terminal LHCSR3 peptide (S/A)V(S/A)GRR(T/A/E)(T/A/E)AAEPQTAAPVAAEDVFAYTKSA. [file TPJ-99-877-s005.pdf]

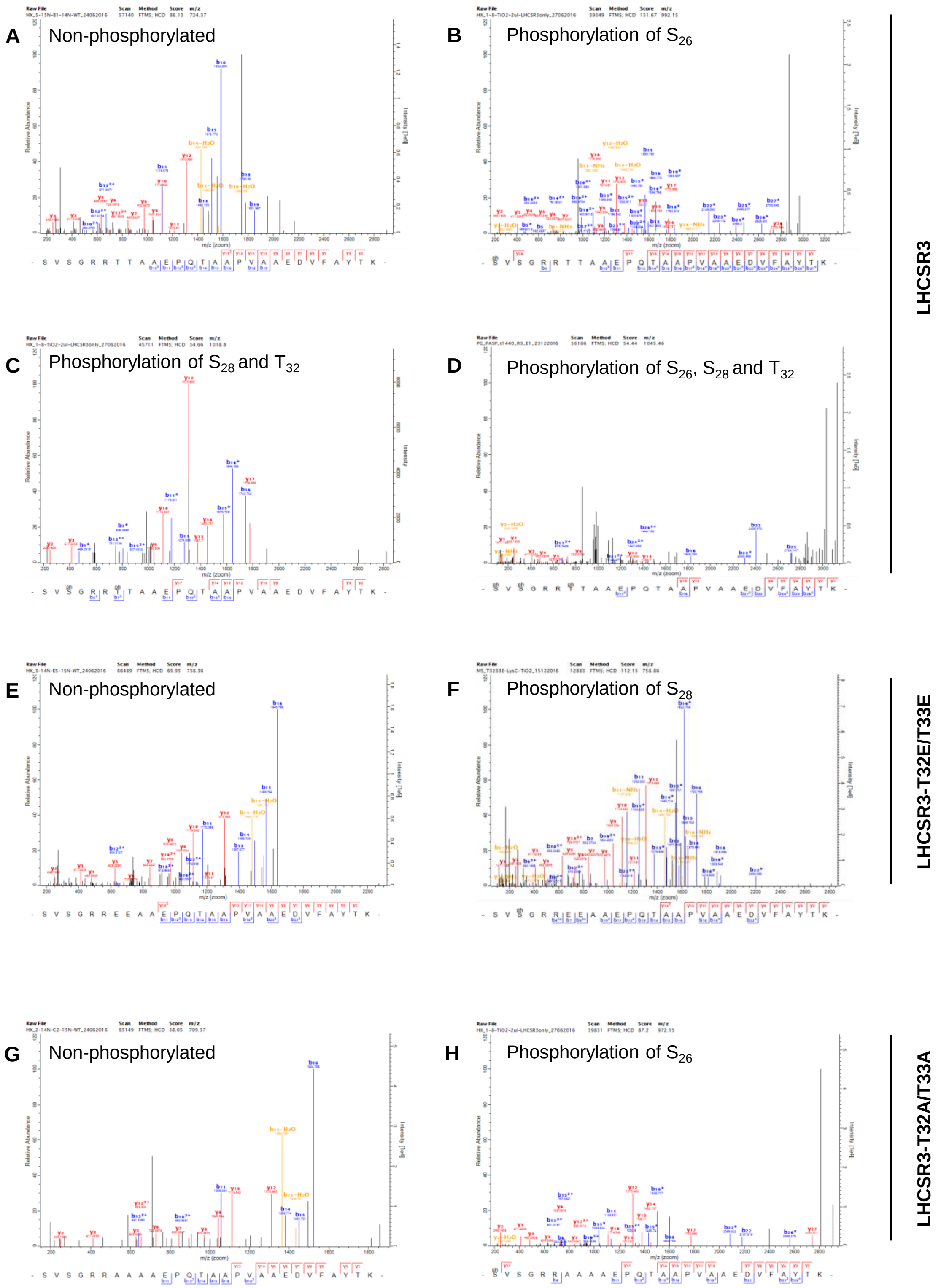

A
Non-phosphorylated
B
Phosphorylation of $S_{26}$
C
Phosphorylation of $S_{28}$ and $T_{32}$
D
Phosphorylation of $S_{26}$, $S_{28}$ and $T_{32}$
LHCSR3
E
Non-phosphorylated
F
Phosphorylation of $S_{28}$
LHCSR3-T32E/T33E
G
Non-phosphorylated
H
Phosphorylation of $S_{26}$
LHCSR3-T32A/T33A

I

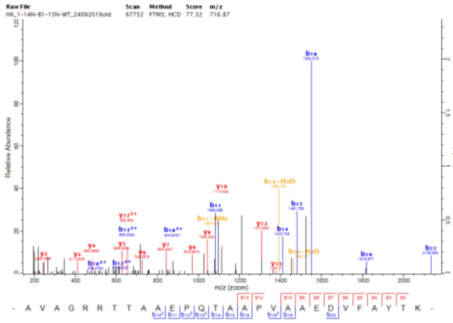


LHCSR3-S26A/S28A

**Figure S5.** Annotated fragmentation spectra of non-phosphorylated and phosphorylated versions of the N-terminal LHCSR3 peptide (S/A)V(S/A)GRR(T/A/E)(T/A/E)AAEPQTAAPVAAEDVFAYTKSA.

**(A-D)** Wildtype LHCSR3.

**(E-F)** LHCSR3-T32E/T33E.

**(G-H)** LHCSR3-T32A/T33A.

**(I)** LHCSR3-S26A/S28A.
