# Supplementary material for: Light‐dependent N‐terminal phosphorylation of LHCSR3 and LHCB4 are interlinked in Chlamydomonas reinhardtii
Source: Plant J. 2019 May 30;99(5):877–94. doi: 10.1111/tpj.14368 (PMC6851877; doi:10.1111/tpj.14368)
Supplement: Supplementary file 6 — Figure S6. Changes in relative protein/peptide abundances and phosphorylation levels in LHCSR3 mutant and rescued strains. [file TPJ-99-877-s006.pdf]

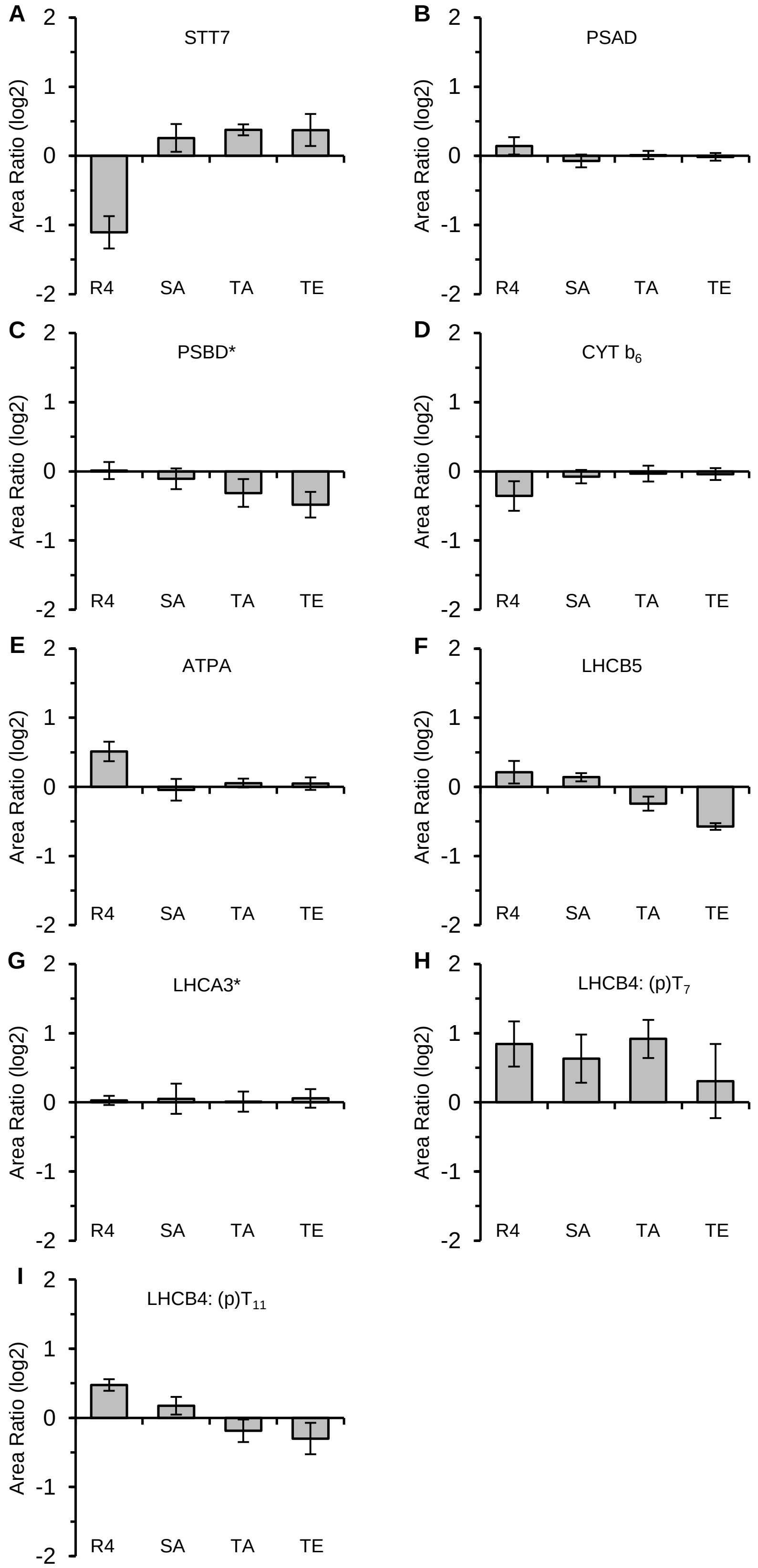
A
STT7
B
PSAD
C
PSBD*
D
CYT b6
E
ATPA
F
LHCB5
G
LHCA3*
H
LHCB4: (p)T7
I
LHCB4: (p)T11
Area Ratio (log2)
R4
SA
TA
TE
2
1
0
-1
-2

Figure S6. Changes in relative protein/peptide abundances and phosphorylation levels in LHCSR3 mutant and rescued strains.

For details regarding experimental conditions and quantification see **Fig. 6**. R4, LHCSR3-R4; SA, LHCSR3-S26A/S28A; TA, LHCSR3-T32A/T33A; TE, LHCSR3-T32E/T33E; *, quantified only in samples treated with trypsin; (p), phosphorylated residue.

(A) STT7 **(B)** Photosystem I reaction center subunit II (PSAD) **(C)** Photosystem II D2 protein (PSBD) **(D)** Cytochrome b_6 **(E)** CF1 ATPase beta-subunit (ATPB) **(F)** LHCB5 **(G)** LHCA3 **(H)** Singly LHCB4 peptide $FP(p)T_7PPGT_{11}QK$. **(I)** Singly LHCB4 peptide $FPT_7PPG(p)T_{11}QK$.
