# Supplementary material for: Light‐dependent N‐terminal phosphorylation of LHCSR3 and LHCB4 are interlinked in Chlamydomonas reinhardtii
Source: Plant J. 2019 May 30;99(5):877–94. doi: 10.1111/tpj.14368 (PMC6851877; doi:10.1111/tpj.14368)
Supplement: Supplementary file 7 — Figure S7. Analysis of the thylakoid membrane composition in strains expressing LHCSR3 with altered N‐terminal phosphorylation sites by sucrose density gradient separation. [file TPJ-99-877-s007.pdf]

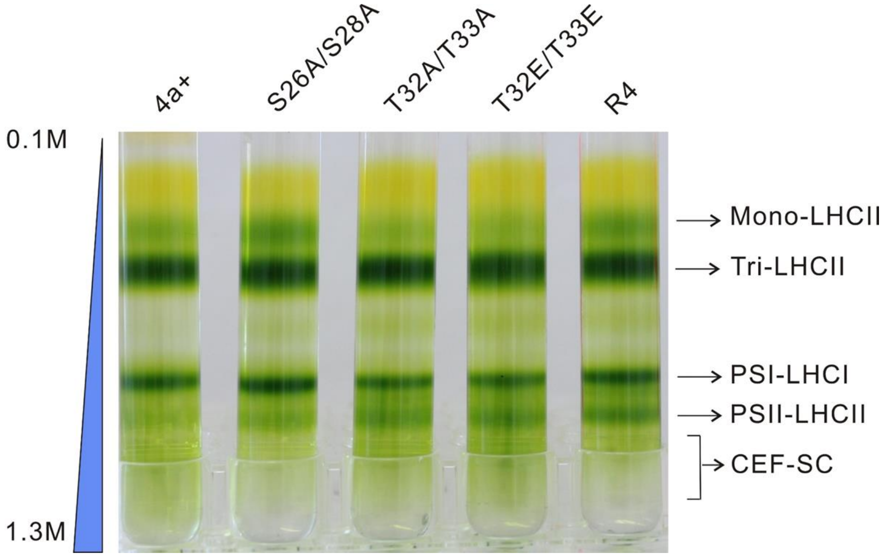

Figure S7. Analysis of the thylakoid membrane composition in strains expressing LHCSR3 with altered N-terminal phosphorylation sites by sucrose density gradient separation.

Thylakoids were isolated from wild type 4a+ and strains expressing LHCSR3 with altered N-terminal phosphosites (S26A/S28A, T32A/T33A, T32E/T33E) or wild typical LHCSR3 (R4) under the control of the constitutively active PSAD promotor. Culures were pre-grown for 24 h under 200 µmol photons m^{-2} s^{-1} high light prior to thylakoid isolation. Solubilization took place in the presence of 1% (w/v) n-dodecyl-α-D-maltoside. Solubilized thylakoid membranes were separated on continuous sucrose density gradients (0.1 M sucrose at the top to 1.3 M sucrose at the bottom), forming a distinct band pattern with monomeric LHCII antenna on top, followed by trimeric LHCII antenna, the PSI-LHCI and the PSII-LHCII complexes, with the putative PSI cyclic electron flow supercomplex (CEF-SC) near the bottom of the gradients.
