# Supplementary material for: Light‐dependent N‐terminal phosphorylation of LHCSR3 and LHCB4 are interlinked in Chlamydomonas reinhardtii
Source: Plant J. 2019 May 30;99(5):877–94. doi: 10.1111/tpj.14368 (PMC6851877; doi:10.1111/tpj.14368)
Supplement: Supplementary file 8 — Figure S8. qE‐dependent NPQ and maximum quantum yield in WT, S26A/S28A, T32A/T33A, T32E/T33E, R10 (equivalent to R4) and npq4. [file TPJ-99-877-s008.pdf]

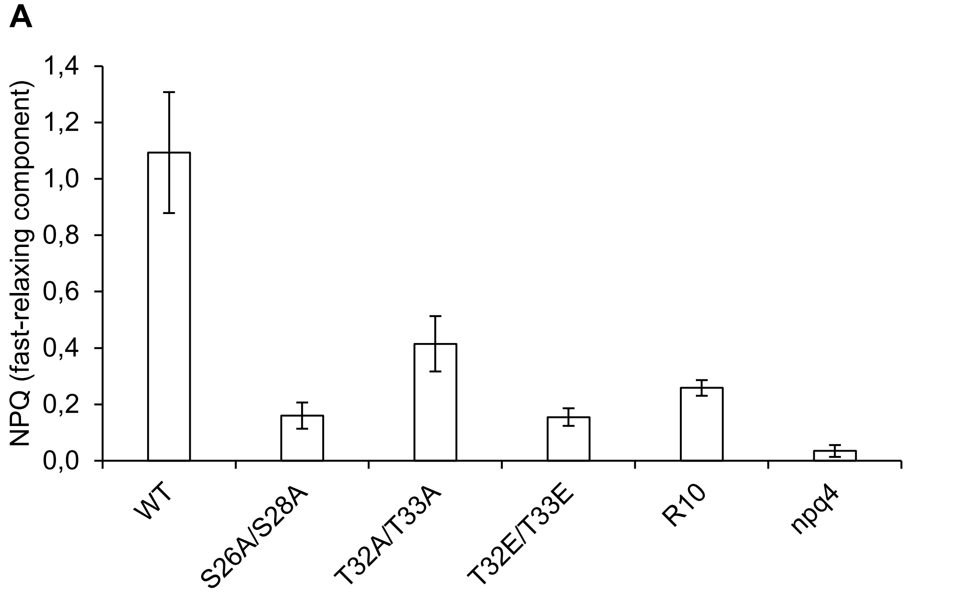

Figure S8. qE-dependent NPQ and maximum quantum yield in WT, S26A/S28A, T32A/T33A, T32E/T33E, R10 (equivalent to R4) and npq4

NPQ was measured after 24 h at 200 µmol photons m^{-2} s^{-1} (A). The maximum quantum yield (Fv/Fm) was measured prior to and following 24 h at 200 µmol photons m^{-2} s^{-1} (B). Prior to the high light exposure, cultures were shifted to HSM and adjusted to 8 µg mL^{-1} chlorophyll. Before the measurements, cells were dark adapted for at least 10 min. The amplitude of the fast-relaxing NPQ component was assessed within the first 30 s of darkness following 3.5 min at 800 µmol photons m^{-2} s^{-1}. Data points represent means ± SEM of 3 biological replicates.
