# Supplementary material for: Light‐dependent N‐terminal phosphorylation of LHCSR3 and LHCB4 are interlinked in Chlamydomonas reinhardtii
Source: Plant J. 2019 May 30;99(5):877–94. doi: 10.1111/tpj.14368 (PMC6851877; doi:10.1111/tpj.14368)
Supplement: Supplementary file 9 — Table S1. Observed phosphopeptides of LHCSR3 (wild type and mutant versions). [file TPJ-99-877-s009.pdf]

Table S1

Table S1. Observed phosphopeptides of LHCSR3 (wildtype and mutant versions)**.**

Peptide identification and determination of phosphorylation sites were performed using MaxQuant. PEP, posterior error probability.

Protein	Positions within Protein	Localization Probability	PEP	Score for Localization	Number of Phospho	Amino Acid	Phospho Site Probabilities
LHCSR3	26	0.84	2.83E-65	129.5	2;3	S	S(0.836)VS(0.623)GRRT(0.411)T(0.129)AAEPQTAAPVAAEDVFAYTK
LHCSR3	28	0.72	5.27E-65	54.441	2;3	S	S(0.763)VS(0.717)GRRT(0.686)T(0.686)AAEPQT(0.149)AAPVAAEDVFAYTK
LHCSR3	32	0.69	1.13E-135	54.441	1;2;3	T	S(0.763)VS(0.717)GRRT(0.686)T(0.686)AAEPQT(0.149)AAPVAAEDVFAYTK
LHCSR3	33	0.94	2.72E-264	202.2	1;2;3	T	RT(0.059)T(0.941)AAEPQTAAPVAAEDVFAYTK
LHCSR3	39	0.33	3.70E-3	40.381	1	T	T(0.333)T(0.333)AAEPQT(0.333)AAPVAAEDVFAYTK
LHCSR3 (T32E/T33E)	28	0.53	1.4E-12	112.15	1	S	S(0.466)VS(0.533)GRREEAAEPQTAAPVAAEDVFAYTK
LHCSR3 (T32A/T33A)	26	0.80	1.9E-67	87.203	1	S	S(0.801)VS(0.195)GRRAAAAEPQT(0.004)AAPVAAEDVFAYTK
LHCSR3 (T32A/T33A)	28	0.44	6.5E-13	54.703	1	S	S(0.441)VS(0.441)GRRAAAAEPQT(0.119)AAPVAAEDVFAYTK
