# Supplementary material for: Light‐dependent N‐terminal phosphorylation of LHCSR3 and LHCB4 are interlinked in Chlamydomonas reinhardtii
Source: Plant J. 2019 May 30;99(5):877–94. doi: 10.1111/tpj.14368 (PMC6851877; doi:10.1111/tpj.14368)
Supplement: Supplementary file 10 — Table S2. LC‐MS/MS parameters for DDA analyses (for generation of spectral libraries and non‐targeted quantification by MS1 feature detection). [file TPJ-99-877-s010.pdf]

Table S2

Table S2. LC-MS/MS parameters for DDA analyses (for generation of spectral libraries and quantification by MS1 feature detection (MaxLFQ)).

	LHCSR3 phosphosite mutants				High light kinetics				
	Trypsin digested samples		LysC digested samples		200 µmol photons $m^{-2} s^{-1}$		500 µmol photons $m^{-2} s^{-1}$		
	non-enriched	enriched	non-enriche	enriched	non-enriched	enriched	non-enriched	enriched	
**Eluent compositions**	Peptide trapping: 0.05% trifluoroacetic acid (TFA) in ultrapure water (A1), 0.05% TFA in 80% acetonitrile (B1)<br>Peptide separation: 0.1% formic acid (FA) in ultrapure water (A2), 0.1% FA in 80% acetonitrile (B2)								**LC parameters**
**Trap Column**	C18 PepMap 100, 300 µM x 5 mm, 5 µm particle size, 100 Å pore size; Thermo Scientific								
**Peptide trapping (eluents A1+B1)**	2.5% B1 at 10 µl/min for 10 min				2.5% B1 at 10 µl/min for 5 min				
**Flow rate**	300 nl/min								
**Gradient for peptide separation**	2.5-30% B2 over 240 min,<br>30-50% B2 over 60 min,<br>50%-99% B2 over 5 min<br>99% B2 over 15 min				2.5-18% B2 over 105 min,<br>18%-32% B2 over 55 min,<br>32%-99% B2 over 5 min,<br>99% B over 20 min		2.5-18% B2 over 65 min,<br>18%-35% B2 over 40 min,<br>35%-99% B2 over 5 min,<br>99% B2 over 20 min		
**Separation column**	Acclaim PepMap C18, 75 µm x 50 cm, 2 µm particle size, 100 Å pore size; Thermo Scientific								
**Resolution at *m/z* 200 (FWHM)**	70,000								**MS1 settings**
**Chromatographic peak width**	30 s				20 s	30 s	20 s	30s	
**AGC target**	1e6								
**Maximum injection time**	50 ms	100 ms	50 ms		30 ms	50 ms	50 ms	100 ms	
**Scan range**	375-1600 *m/z*				350-1600 *m/z*		350-1500 *m/z*		
**TopN**	12								**MS2 settings**
**Resolution at *m/z* 200 (FWHM)**	17,500								
**Isolation window**	1.5 *m/z*								
**AGC target**	5e4			1e5	5e4				
**Maximum injection time**	55 ms	125 ms	55 ms		55 ms	110 ms	55 ms	125 ms	
**Normalized collision energy (NCE)**	27								
**Minimum AGC target**	2e2	1.25e3	5e2		5.5e2	1e3	5.5e2	1.25e3	
**Intensity threshold**	3.6e3	1e4	9.1e3		1e4	9.1e3	1e4		
**Charge exclusion**	unassigned,1, >7				unassigned,1, >4		unassigned,1, >5		
**Dynamic exclusion**	60 s				auto		auto	30 s	
