# Supplementary material for: Light‐dependent N‐terminal phosphorylation of LHCSR3 and LHCB4 are interlinked in Chlamydomonas reinhardtii
Source: Plant J. 2019 May 30;99(5):877–94. doi: 10.1111/tpj.14368 (PMC6851877; doi:10.1111/tpj.14368)
Supplement: Supplementary file 11 — Table S3. LC‐MS/MS parameters for PRM analyses. [file TPJ-99-877-s011.pdf]

Table S3

Table S3. LC-MS/MS parameters for PRM analyses. *, Single Ion Monitoring (SIM) data was acquired in parallel to PRM data, but was excluded from further analyses.

	**LHCSR3 phosphosite mutants***		**High light kinetics**		
	Trypsin digested samples	LysC digested samples	200 µmol photons $m^{-2} s^{-1}$	500 µmol photons $m^{-2}s^{-1}$	
**Eluent compositions**	Peptide trapping: 0.05% trifluoroacetic acid (TFA) in ultrapure water (A1), 0.05% TFA in 80% acetonitrile (B1)<br>Peptide separation: 0.1% formic acid (FA) in ultrapure water (A2), 0.1% FA in 80% acetonitrile (B2)				**LC parameters**
**Trap Column**	C18 PepMap 100, 300 µM x 5 mm, 5 µm particle size, 100 Å pore size; Thermo Scientific				
**Peptide trapping (eluents A1+B1)**	2.5% B1 at 10 µl/min for 10 min		2.5% B1 at 10 µl/min for 5 min		
**Separation Column**	Acclaim PepMap C18, 75 µm x 50 cm, 2 µm particle size, 100 Å pore size; Thermo Scientific		Acclaim PepMap C18, 75 µm x 15 cm, 2 µm particle size, 100 Å pore size; Thermo Scientific	Acclaim PepMap C18, 75 µm x 50 cm, 2 µm particle size, 100 Å pore size; Thermo Scientific	
**Gradient for peptide separation (eluents A2+B2)**	2.5-30% B2 over 240 min, 30-50% B2 over 60 min, 50%-99% B2 over 5 min 99% B2 over 15 min		2.5-15% B2 over 47 min, 15%-34% B2 over 33 min, 34%-99% B2 over 5 min, 99% B over 5 min	2.5-18% B2 over 65 min, 18%-35% B2 over 40 min, 35%-99% B2 over 5 min, 99% B2 over 20 min	
**Flow rate**	300 nl/min				
**Chromatographic peak width**	15 s				**MS settings (PRM)**
**Resolution (FWHM) at m/z 200**	70,000	35,000	35,000		
**AGC target**	1e5		5e4		
**Maximum injection time**	480 ms	240 ms	240 ms		
**Isolation window**	2 m/z	3 m/z	2 m/z		
**Isolation offset**	0 m/z	1 m/z	0.5 m/z		
**Normalized collision energy (NCE)**	27				
