# Supplementary material for: Light‐dependent N‐terminal phosphorylation of LHCSR3 and LHCB4 are interlinked in Chlamydomonas reinhardtii
Source: Plant J. 2019 May 30;99(5):877–94. doi: 10.1111/tpj.14368 (PMC6851877; doi:10.1111/tpj.14368)
Supplement: Supplementary file 16 [file TPJ-99-877-s016.docx]

**Supporting Info Legends**

**Figure S1**. Western Blot analysis of whole cell extracts from cultures exposed to 200 μmol photons m^‑2^ s^‑1^ or 500 μmol photons m^-2^ s^-1^ high light. Samples were taken after 0 h, 1 h, 4 h and 24 h. For SDS-PAGE, whole cell samples were adjusted to equal protein amounts.

**Figure S2.** Changes in the abundance of singly and multiply phosphorylated variants of the LHCSR3 peptide S_26_VS_28_GRRT_32_T_33_AAEPQTAAPVAAEDVFAYTK in response to different light conditions.

Algal cultures were exposed to 200 μmol photons m^-2^ s^-1^ **(A, C, E)** or 500 μmol photons m^-2^ s^-1^ **(B, D, F)** high light in HS medium and samples were taken at the indicated timepoints. Label-free quantification was performed as described in Figure 1.

**(A, B)** Singly phosphorylated peptide S_26_VS_28_GRRT_32_T_33_AAEPQTAAPVAAEDVFAYTK

**(C, D)** Same peptide as in (A,B), doubly phosphorylated.

**(E, F)** Same peptide as in (A,B), triply phosphorylated.

Data represent mean +/- standard deviation (200 μmol photons m^-2^ s^-1^: *n*=4; 500 μmol photons m^-2^ s^-1^: *n*=3 (0h: *n*=2)). Numbers in parentheses indicate the number of data points if low abundances did not permit quantification in all replicates. Welch’s t-test (unpaired, two-tailed) was used to analyse the data: Values labelled with identical letters, or no letters, don’t show statistically significant differences (p > 0.05).

Only kinetics of protein abundance changes but not absolute protein or peptide levels are comparable between the two high light experiments, since samples were analysed with different LC-MS configurations.

**Figure S3.** Immunoblot analysis of solubilized LHCSR3-T32E/T33E thylakoids separated by sucrose density gradient centrifugation and analysis of phosphorylation dependent running behavior of LHCSR3 in SDS-PAGE.

**(A)** Abundance of PSBA, LHCSR3 and PSAD in sucrose density gradient (SDG) fractions obtained from a strain expressing LHCSR3-T32E/T33E. Thylakoids were isolated after 24 h at 200 μmol photons m^‑2^ s^‑1^. Prior to the high light exposure, cultures were shifted to autotrophic medium (HSM) and adjusted to 4 μg mL^-1^ chlorophyll (chl) concentration. Isolated thylakoids were solubilized with 1% n-dodecyl-α-D-maltoside followed by SDG centrifugation. Samples for immunoblots were adjusted to equal volume (100 μL of each SDG fraction). The figure shows two independent experiments. The lower, bold black bar indicates the position of the 25 kDa band of the molecular weight marker. The upper black bar indicates the running height of the upper LHCSR3 band.

**(B)** Abundance of LHCSR3 in thylakoids treated with calf intestinal alkaline phosphatase (CIAP). Thylakoids were isolated from 4A+ and strains expressing LHCSR3-T32E/T33E, -T32A/T33A or wild type LHCSR3 (R10, equivalent to strain R4) under the constitutive PSAD promotor, after 24 h at 200 μmol photons m^-2^ s^-1^. Prior to the high light exposure, cultures were shifted HSM and adjusted to 4 μg mL^-1^ chl concentration. Isolated thylakoids were adjusted to equal chl concentration (120 μg mL^-1^) and treated with CIAP as indicated. 3 μg chl (100%) and 1.5 μg chl (50%) were used for SDS-PAGE and Western Blots. ATPB was used as a loading control. The bold, lower black bar indicates the position of the 25 kDa band of the molecular weight marker. The upper black bar indicates the running height of the upper LHCSR3 band.

**Figure S4.** Immunoblot analysis of the distribution of PSII, PSI and LHCSR3 across sucrose density gradients.

Thylakoid membranes were isolated from isolated from wild type 4a+ and strains expressing LHCSR3

with altered N-terminal phosphosites (S26A/S28A, T32A/T33A) or wild typical LHCSR3 (R4) under the

control of the constitutively active PSAD promotor. Prior to the thylakoid isolation, strains were grown in

autotrophic conditions at 200 μmol photons m^-2^ s^-1^ for 24 h. After solubilization by 1 % n-dodecyl-α-D-maltoside and separation on sucrose density gradients, gradients were separated into 29 fractions, beginning with high sucrose density fractions at the bottom. Fractions were further analyzed by SDS-PAGE and Immunoblot. Samples for SDS-PAGE were adjusted to the equal volume.

**(A)** Fractions 2-15, analyzed using antibodies targeting PSII subunit PSBA, PSI subunit PSAD and

LHCSR3.

**(B)** Fractions 16-29 analyzed using antibodies targeting LHCSR3.

**Figure S5.** Annotated fragmentation spectra of non-phosphorylated and phosphorylated versions of the N-terminal LHCSR3 peptide (S/A)V(S/A)GRR(T/A/E)(T/A/E)AAEPQTAAPVAAEDVFAYTKSA.

**(A-D)** Wildtype LHCSR3.

**(E-F)** LHCSR3-T32E/T33E.

**(G-H)** LHCSR3-T32A/T33A.

**(I)** LHCSR3-S26A/S28A.

**Figure S6.** Changes in relative protein/peptide abundances and phosphorylation levels in LHCSR3 mutant and rescued strains.

For details regarding experimental conditions and quantification see Fig. 6. R4, LHCSR3-R4; SA, LHCSR3-S26A/S28A; TA, LHCSR3-T32A/T33A; TE, LHCSR3-T32E/T33E; *, quantified only in samples treated with trypsin; (p), phosphorylated residue.

**(A)** STT7. **(B)** Photosystem I reaction center subunit II (PSAD). **(C)** Photosystem II D2 protein (PSBD). **(D)** Cytochrome b_6_. **(E)** CF1 ATPase beta-subunit (ATPB). **(F)** LHCB5. **(G)** LHCA3. **(H)** Singly LHCB4 peptide FP(p)T_7_PPGT11QK. **(I)** Singly LHCB4 peptide FPT_7_PPG(p)T_11_QK.

**Figure S7.** Analysis of the thylakoid membrane composition in strains expressing LHCSR3 with altered N-terminal phosphorylation sites by sucrose density gradient separation.

Thylakoids were isolated from wild type 4a+ and strains expressing LHCSR3 with altered N-terminal

phosphosites (S26A/S28A, T32A/T33A, T32E/T33E) or wild typical LHCSR3 (R4) under the control of the constitutively active PSAD promotor. Cultures were pre-grown for 24 h under 200 μmol photons m^‑2^s^‑1^ high light prior to thylakoid isolation. Solubilization took place in the presence of 1% (w/v) n-dodecyl-α-D-maltoside. Solubilized thylakoid membranes were separated on continuous sucrose density gradients (0.1 M sucrose at the top to 1.3 M sucrose at the bottom), forming a distinct band pattern with monomeric

LHCII antenna on top, followed by trimeric LHCII antenna, the PSI-LHCI and the PSII-LHCII complexes, with the putative PSI cyclic electron flow supercomplex (CEF-SC) near the bottom of the gradients.

**Figure S8.** qE-dependent NPQ and maximum quantum yield in WT, S26A/S28A, T32A/T33A, T32E/T33E, R10 (equivalent to R4) and npq4.

NPQ was measured after 24 h at 200 μmol photons m^-2^ s^-1^ **(A)**. The maximum quantum yield (Fv/Fm) was measured prior to and following 24 h at 200 μmol photons m^-2^ s^-1^ **(B)**. Prior to the high light exposure, cultures were shifted to HSM and adjusted to 8 μg mL^-1^ chlorophyll. Before the measurements, cells were dark adapted for at least 10 min. The amplitude of the fast-relaxing NPQ component was assessed within the first 30 s of darkness following 3.5 min at 800 μmol photons m^-2^ s^-1^. Data points

represent means ± SEM of 3 biological replicates.

**Table S1.** Observed phosphopeptides of LHCSR3 (wildtype and mutant versions)**.**

Peptide identification and determination of phosphorylation sites were performed using MaxQuant. PEP, posterior error probability.

**Table S2.** LC-MS/MS parameters for DDA analyses (for generation of spectral libraries and

quantification by MS1 feature detection (MaxLFQ)).

**Table S3.** LC-MS/MS parameters for PRM analyses. *, Single Ion Monitoring (SIM) data was

acquired in parallel to PRM data, but was excluded from further analyses.
